# Modulation of gene expression in heart and liver of hibernating black bears *(Ursus americanus)*

**DOI:** 10.1186/1471-2164-12-171

**Published:** 2011-03-31

**Authors:** Vadim B Fedorov, Anna V Goropashnaya, Øivind Tøien, Nathan C Stewart, Celia Chang, Haifang Wang, Jun Yan, Louise C Showe, Michael K Showe, Brian M Barnes

**Affiliations:** 1 Institute of Arctic Biology, University of Alaska Fairbanks, Fairbanks, AK 99775, USA; 2 Systems Biology Division, the Wistar Institute, Philadelphia, PA 19104, USA; 3 CAS-MPG Partner Institute for Computational Biology, Shanghai Institutes of Biological Sciences, 320 Yue Yang Road, Shanghai, 200031, PR China

## Abstract

**Background:**

Hibernation is an adaptive strategy to survive in highly seasonal or unpredictable environments. The molecular and genetic basis of hibernation physiology in mammals has only recently been studied using large scale genomic approaches. We analyzed gene expression in the American black bear, *Ursus americanus*, using a custom 12,800 cDNA probe microarray to detect differences in expression that occur in heart and liver during winter hibernation in comparison to summer active animals.

**Results:**

We identified 245 genes in heart and 319 genes in liver that were differentially expressed between winter and summer. The expression of 24 genes was significantly elevated during hibernation in both heart and liver. These genes are mostly involved in lipid catabolism and protein biosynthesis and include RNA binding protein motif 3 (*Rbm3*), which enhances protein synthesis at mildly hypothermic temperatures. Elevated expression of protein biosynthesis genes suggests induction of translation that may be related to adaptive mechanisms reducing cardiac and muscle atrophies over extended periods of low metabolism and immobility during hibernation in bears. Coordinated reduction of transcription of genes involved in amino acid catabolism suggests redirection of amino acids from catabolic pathways to protein biosynthesis. We identify common for black bears and small mammalian hibernators transcriptional changes in the liver that include induction of genes responsible for fatty acid β oxidation and carbohydrate synthesis and depression of genes involved in lipid biosynthesis, carbohydrate catabolism, cellular respiration and detoxification pathways.

**Conclusions:**

Our findings show that modulation of gene expression during winter hibernation represents molecular mechanism of adaptation to extreme environments.

## Background

Hibernation is an adaptive strategy involving regulated metabolic suppression used by taxonomically diverse mammalian species to conserve energy during periods of low food availability [1,2]. The molecular and genetic basis of hibernation physiology in mammals has only recently been studied using large scale genomic approaches. Genome-wide approaches reveal the significance of transcriptional changes by identifying functional groups of co-regulated differentially expressed genes within metabolic and signaling pathways. Recent studies of differential gene expression at the genomic scale on several species of small hibernating mammals have detected expression changes for hundreds of genes and identified groups of co-regulated genes involved in carbohydrate and lipid metabolism, detoxification, and molecular transport when comparing animals sampled in different stages during hibernation and non-hibernating periods [3-6].

The black bear (*Ursus americanus*) provides a distinct example of hibernation in mammals. Unlike small animals (<5 kg) such as hamsters, ground squirrels, and marmots, that reach body temperatures near 0°C during torpor, hibernating black bears (30-200 kg) maintain core body temperatures above 30°C [7] and remain capable of moving throughout hibernation [8]. Bears do not eat, drink, defecate, or urinate throughout a 3-6 month hibernation season [9]. Heart rate in hibernating bears decreases from 60 bpm to 16 bpm [10]. Metabolic rate in hibernating bears is reduced by 20-50%, and it takes several weeks after emergence for metabolic rate to return to normal levels [7].

We recently developed a collection of expressed sequence tags (ESTs) specifically for *U. americanus*, and used the resulting pilot version of microarray with 3,200 probes to detect gene expression differences in liver and skeletal muscle sampled during hibernation compared to in animals sampled during summer [11]. We found a highly significant enrichment during hibernation of the protein biosynthesis category by over-expressed genes in both liver and skeletal muscle. These results demonstrated efficiency of our approach to generating genomic resources and fabricating custom microarrays for non model species. We, therefore, extended gene discovery study and generated additional 32,000 ESTs for the black bear [12]. In the present study, we used a more representative set of 9,600 cDNA probes to identify more co-regulated functional groups of differentially expressed genes in liver. To obtain the first insight into transcriptional changes in heart of hibernating bears, we used the complete set of 12,800 probes available for the black bear [12]. We focus on pathway analysis identifying functional groups of co-regulated genes rather than on expression of individual genes to assess the biological significance of transcriptional changes associated with hibernation in black bears. Liver and heart play an important role in homeostasis and transcriptional changes in these vital organs have been recently reported for small hibernators [4,6]. To investigate any general patterns in transcriptional profiles during mammalian hibernation, we compare functional groups of differentially expressed genes detected in hibernating bears to changes reported for small mammalian hibernators.

## Results

### Body temperature, metabolism and heart rate

As previously reported, hibernating bears had core body temperatures of 34.2 ± 0.5°C (mean ± SD, n = 6) and minimum rates of oxygen consumption of 0.083 ± 0.008 ml g^-1 ^h^-1 ^(n = 5), when measured over at least a 0.5 h interval 2-9 hours before euthanasia [11]. Heart rate (HR) derived by counting beats within a 240 s on ECG recording during this period was 14.4 ± 2.4 b/min. (mean ± SD, n = 6) and showed pronounced respiratory related sinus arrhythmia. In four hibernating bears for which post-immobilization measurements were made, body temperature decreased from 34.2 ± 0.7°C to 33.3 ± 1.1°C (p < 0.05) and HR increased from 13.4 ± 2.2 to 82.9 ± 22.8 b/min (p < 0.05). Sinus arrhythmia was absent in anesthetized bears. Bears lost 4.7 ± 0.8% (n = 6) of their body mass per month during the 4-5 month hibernation period. Metabolic rates measured in two fasted and anesthetized summer active bears were 0.252 and 0.213 ml g^-1 ^h^-1 ^and averaged 0.233 ml g^-1 ^h^-1^; body temperatures were 37.18 and 37.13°C [11] and HR was 102.3 and 97.2 b/min and averaged 99.7 b/min. Immediately prior to tissue sampling, metabolic rate of hibernating bears was 36-49%, and body temperature averaged 3.75°C lower compared to values in summer bears [11] while HR was at about the same level due to the effect of anesthesia.

### Difference in gene expression

For heart, signals from 2,594 of 3,200 probes (81.1%) on the first bear array showed median intensities that were above the level of two backgrounds, whereas 6,860 of 9,600 (71.5%) probes showed significant signals on the second bear array (see Material and Methods for the array description). In order to define genes that were differentially expressed in hibernating compared to summer active bears, we used P < 0.01 and log_2_FC > 0.5 where FC is fold change, the mean expression value in the hibernating bears divided by the mean expression value in the summer active bears, as the cutoff for differentially expressed genes (as described in Methods). A total of 245 genes, 3.3% of all unique genes with significant signals (7,359 genes), were differentially expressed in heart during hibernation (Additional file 1, Table S1). The maximal change of 7.28 fold (log_2_FC = 2.86) was detected for RNA binding protein motif 3 (*Rbm3*), but most genes (74%) demonstrated moderate changes in expression less than two fold (log_2_FC < 1). Of the significantly differentially expressed genes, we identified 183 (75%) that were over-expressed and 62 (25%) genes that were under-expressed in heart during hibernation.

For liver, 6,860 of 9,600 (71.5%) probes showed significant signals on the second bear array, and 319 genes of these, 6.2% of all unique genes with significant signal (5,092 genes), were differentially expressed in liver during hibernation (Additional file 1, Table S1). There were 165 (52%) significantly over-expressed genes and 154 (48%) under-expressed genes in liver sampled during hibernation compared to in summer. Phosphoenolpyruvate carboxykinase (*Pck1*) showed the largest positive expression change of 10.91 fold (log_2_FC = 3.448), and aldehyde dehydrogenase (*Aldh1l1*) was 17.94 fold down regulated (log_2_FC = -4.168), but most genes (74%) demonstrated modest expression changes that did not exceed two fold (|log_2_FC| < 1).

**Figure 1:**
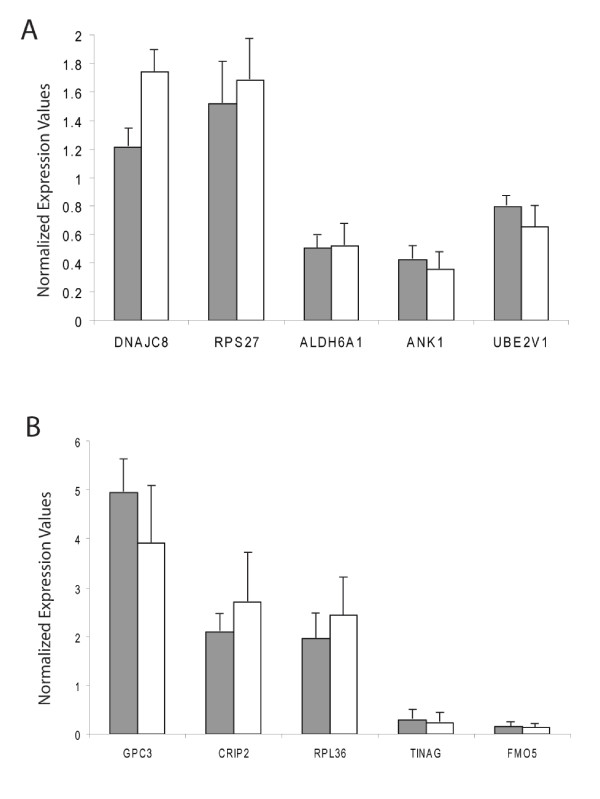
**Selection of genes differentially expressed during hibernation in heart (A) and liver (B) tissue in black bears**. Expression values are normalized to the mean in summer active animals. Solid bars show expression values obtained in real-time PCR, open bars in microarray experiments, error bars are SDs.

To validate the microarray results, we conducted quantitative real-time PCR tests for 32 randomly selected genes that were identified as differentially expressed by the array hybridizations. Expression changes of 28 (87.5%) out of 32 genes identified on the array were confirmed by the RT PCR tests (Table 1) with significant positive correlation (r = 0.83, p < 0.001) between fold change values of supported genes (Table 1, Figure 1). High consistency between microarray experiments and RT PCR tests is in agreement with the mean false discovery rate of 12% for the list of differentially expressed genes.

**Table 1:** Gene expression differences tested by microarray and real-time PCR in bear heart and liver

Gene Symbol	Gene Name	RT-PCR		Microarray	
		
		P	**log**_**2**_**FC**	P	**log**_**2**_**FC**
HEART					
*Acadvl*	Acyl-Coenzyme A dehydrogenase, very long chain	0.034	0.122	<0.001	0.747
*Dnajc8*	DnaJ (Hsp40) homolog, subfamily C, member 8	0.008	0.270	<0.001	0.698
*Gapdh*	Glyceraldehyde-3-phosphate dehydrogenase	**0.140**	n/a	**0.008**	0.620
*Mbip*	MAP3K12 binding inhibitory protein 1	**0.580**	n/a	**0.001**	0.884
*Rbm3*	RNA binding motif protein 3	<0.001	2.349	<0.001	2.863
*Rpl35a*	Ribosomal protein L35a	0.009	0.550	<0.001	1.857
*Rpl7*	Ribosomal protein L7	0.004	0.533	<0.001	1.243
*Rps27*	Ribosomal protein S27	0.009	0.600	0.001	0.720
*Adk*	Adenosine kinase	0.015	-0.164	0.006	-0.534
*Aldh6a1*	Aldehyde dehydrogenase 6 family, member A1	<0.001	-0.974	0.001	-0.893
*Ank1*	Ankyrin 1, erythrocytic	<0.001	-1.234	<0.001	-1.440
*Gstz1*	Glutathione transferase zeta 1	0.031	-0.343	0.001	-0.605
*Ube2v1*	Ubiquitin-conjugating enzyme E2 variant 1	0.001	-0.327	0.004	-0.600
LIVER					
*Gpc3*	Glypican 3	<0.001	2.307	<0.001	1.965
*Rplp2*	60S acidic ribosomal protein P2	<0.001	1.378	0.003	1.627
*Cmtm8*	CKLF-like MARVEL transmembrane domain-containing protein 8	**0.170**	n/a	**<0.001**	1.525
*Pc*	Pyruvate carboxylase	**0.130**	n/a	**0.002**	1.390
*Crip2*	Cysteine-rich protein 2	<0.001	1.064	0.001	1.285
*A2m*	Alpha 2 macroglobulin	0.003	1.287	0.004	1.249
*Rpl36*	60S ribosomal protein L36	0.002	0.963	0.004	1.223
*Brp44l*	Brain protein 44-like protein	0.002	1.638	0.009	1.198
*Naca*	Nascent-polypeptide-associated complex alpha polypeptide variant	<0.001	0.714	0.001	1.182
*Fgfr2*	Fibroblast growth factor receptor 2 precursor	<0.001	1.055	<0.001	1.130
*Tbca*	Tubulin cofactor a	0.001	0.579	0.001	1.122
*Rps6*	Ribosomal protein S6	0.001	0.677	0.010	1.079
*Comt*	Catechol-O-methyltransferase	<0.001	-0.741	0.001	-1.817
*Arg1*	Arginase	0.016	-1.418	0.009	-2.050
*Phyh*	Phytanoyl-CoA 2-hydroxylase	0.002	-1.328	0.004	-2.105
*Tinag*	Tubulointerstitial nephritis antigen	<0.001	-1.822	<0.001	-2.141
*Ugt3a2*	UDP glycosyltransferase 3 family, polypeptide A2	<0.001	-4.034	<0.001	-2.949
*Fmo5*	Flavin-containing monooxygenase 5	0.001	-2.631	0.002	-3.002
*Pitrm1*	Pitrilysin metalloprotease 1	0.027	-0.421	0.001	-3.360

Inconsistent significance levels for some genes are in bold.

The expression of 33 genes was changed in common in both liver and heart (Table 2) of which 24 genes were elevated in both tissues. RNA binding motif protein 3 (*Rbm3*) showed high positive expression change in both liver and heart during hibernation. Most genes (70%) up regulated in both tissues are involved in lipid catabolism (3 genes) and protein biosynthesis (14 genes, Table 2). Among five genes down regulated in both heart and liver, three genes are involved in amino acid catabolism. Five genes demonstrated transcriptional changes in opposite directions in liver and heart.

**Table 2:** Differentially expressed genes shared between liver and heart in hibernating bears

Gene Symbol	Gene Name	P, Liver	**log**_**2**_**FC,** Liver	P, Heart	**log**_**2**_**FC, ** Heart
*Fatty Acid Catabolism (4 genes)*					
*Aarsd1*	Alanyl-tRNA synthetase domain containing 1	0.006	0.638	0.006	0.838
*Acadm*	Medium-chain specific acyl-CoA dehydrogenase, mitochondrial precursor	0.001	0.510	<0.001	1.138
*Acadvl*	Acyl-Coenzyme A dehydrogenase, very long chain	0.001	0.842	<0.001	0.747
*Hadha*	Trifunctional enzyme alpha subunit, mitochondrial precursor	0.007	0.586	0.008	0.556
*Protein Biosynthesis (13 genes)*					
*Rps18*	40S ribosomal protein S18	0.001	0.899	0.002	1.144
*Rps29*	Ribosomal protein S29	0.006	0.802	0.001	0.843
*Rbm3*	RNA binding motif protein 3	<0.001	2.681	<0.001	2.863
*Rpl18a*	60S ribosomal protein L18a	<0.001	0.606	0.002	0.607
*Rpl22*	Ribosomal protein L22	0.006	1.398	0.004	0.923
*Rpl24*	Ribosomal protein L24	0.005	0.935	0.003	0.725
*Rpl27a*	Ribosomal protein L27a-like	0.004	1.349	0.008	0.927
*Rpl30*	Ribosomal protein L30	0.004	0.862	0.002	1.010
*Rpl35a*	60S ribosomal protein L35a	0.001	0.937	<0.001	1.857
*Rpl7*	60S ribosomal protein L7	0.008	0.767	<0.001	1.243
*Rplp2*	60S acidic ribosomal protein P2	0.003	1.627	0.001	0.732
*Rps23*	Ribosomal protein S23	<0.001	1.213	0.001	1.793
*Rps24*	Ribosomal protein S24	0.008	0.802	0.002	1.000
*Amino Acid Catabolic Processes (3 genes)*					
*Aldh6a1*	Aldehyde dehydrogenase 6 family, member A1	0.006	-1.086	0.001	-0.893
*Got1*	Aspartate aminotransferase	<0.001	-1.014	0.002	-0.644
*Lap3*	Leucine aminopeptidase 3 protein	0.008	-0.943	0.001	-0.771
*Other GO Categories (13 genes)*					
*Cd2bp2*	CD2 (cytoplasmic tail) binding protein 2	0.005	0.981	0.008	0.614
*Cdk2*	Cyclin-dependent kinase 2	0.001	0.755	0.005	0.827
*Cd2bp2*	CD2 (cytoplasmic tail) binding protein 2	0.005	0.981	0.008	0.614
*Hnrnpa1*	Heterogeneous nuclear ribonucleoprotein A1	0.004	1.418	0.006	1.048
*St7*	Suppression of tumorigenicity 7	0.001	0.909	0.003	0.965
*Tinp1*	TGF-beta-inducible nuclear protein 1	0.010	0.678	<0.001	0.813
*Cyp1a2*	Cytochrome P450, family 1, subfamily A, polypeptide 2	0.007	-2.290	0.005	-0.594
*Phyh*	Phytanoyl-CoA 2-hydroxylase	0.004	-2.105	0.008	-0.865
*Gbe1*	Glycogen branching enzyme	0.007	-0.641	0.002	0.838
*Glrx3*	Glutaredoxin-3	0.004	-0.595	0.001	1.011
*Kif21a*	Kinesin family member 21A	0.010	1.000	0.009	-0.504
*Mnat1*	Menage a trois homolog 1, cyclin H assembly factor	0.007	-0.675	0.006	1.067
*Ppm1k*	Protein phosphatase 1K	0.005	1.107	0.007	-0.615

### Pathway analysis

The Gene Ontology analysis revealed a highly significant enrichment of the protein biosynthesis (translation) category in the biological processes and the RNA binding in the molecular function by over-expressed genes in both heart and liver during hibernation (Tables 3, 4). Four additional categories demonstrated significant enrichment in liver: up regulated genes in the fatty acid beta-oxidation pathway and under expressed genes in amino acid catabolism, cholesterol metabolism and cellular respiration categories during hibernation. The molecular function categories with significantly elevated proportion of down regulated genes in liver included catalytic processes: oxidoreductase and transaminase activities (Table 3).

**Table 3:** Gene Ontology categories significantly enriched with differentially expressed genes

GO Category	Total genes on array	Changed genes	Enrichment	FDR
*Biological Process*				
HEART				
Translation (GO:0006412)	319	32↑	3.334	<0.001
LIVER				
Fatty acid beta-oxidation (GO:0006635)	22	5↑	6.805	0.036
Translation (GO:0006412)	266	28↑	3.152	<0.001
Amino acid catabolic process (GO:0009063)	36	12↓	10.202	<0.001
Cholesterol metabolic process (GO:0008203)	34	7↓	5.951	<0.001
Cellular respiration (GO:0045333)	47	7↓	4.559	0.024
*Molecular Function*				
HEART				
RNA Binding (GO:0003723)	455	35↑	2.500	<0.001
LIVER				
RNA Binding (GO:0003723)	362	28↑	2.326	<0.001
Transaminase activity (GO:0008483)	10	5↓	15.468	0.001
Oxidoreductase activity, acting on the aldehyde or oxo group of donors (GO:0016903)	20	6↓	9.281	0.001
Monooxygenase activity (GO:0004497)	27	7↓	8.020	0.001
Electron carrier activity (GO:0009055)	98	11↓	3.472	0.003

Arrows indicate direction of gene regulation in hibernating black bears. FDR is false discovery rate.

**Table 4:** Genes in significant Gene Ontology categories of the biological processes

GO category	Gene Name	Gene Symbol	P value	Fold Change **(Log**_**2**_**FC)**
HEART				
Up-regulated				
Translation (GO:0006412)	RNA binding motif protein 3	***Rbm3***	<0.001	2.863
	Ribosomal protein L35A	***Rpl35a***	<0.001	1.857
	Ribosomal protein S23	***Rps23***	0.001	1.793
	Ribosomal protein L7A	*Rpl7a*	0.002	1.287
	Ribosomal protein L7	***Rpl7***	<0.001	1.243
	Eukaryotic translation elongation factor 1 beta 2	*Eef1b2*	0.001	1.187
	Ribosomal protein S3	***Rps3***	0.005	1.166
	Ribosomal protein L10A	*Rpl10a*	0.003	1.156
	Eukaryotic translation initiation factor 5A	*Eif5a*	<0.001	1.147
	Ribosomal protein S15A	*Rps15a*	0.008	1.116
	Ribosomal protein L27	***Rpl27***	0.003	1.099
	Lysyl-trna synthetase	*Kars*	<0.001	1.075
	Ribosomal protein S4, X-linked	*Rps4x*	0.001	1.018
	Ribosomal protein S13	*Rps13*	0.003	1.012
	Ribosomal protein L30	***Rpl30***	0.002	1.010
	Ribosomal protein S24	***Rps24***	0.002	1.000
	Ribosomal protein, large, P0	*Rplp0*	0.001	0.936
	Ribosomal protein L27A	***Rpl27a***	0.008	0.927
	Ribosomal protein L22	***Rpl22***	0.004	0.923
	Ribosomal protein L36A-like	*Rpl36al*	0.005	0.917
	Ubiquitin A-52 residue ribosomal protein fusion product 1	*Uba52*	0.009	0.915
	Eukaryotic translation initiation factor 3, subunit G	*Eif3g*	0.001	0.910
	Ribosomal protein L5	*Rpl5*	0.002	0.900
	Eukaryotic translation initiation factor 3, subunit F	*Eif3f*	0.003	0.884
	Ribosomal protein L10A	*Rpl10*	0.008	0.863
	Alanyl-trna synthetase domain containing 1	***Aarsd1***	0.006	0.838
	Ribosomal protein, large, P2	***Rplp2***	0.001	0.732
	Ribosomal protein L24	***Rpl24***	0.003	0.725
	Ribosomal protein S27	*Rps27*	0.001	0.720
	Eukaryotic translation initiation factor 2, subunit 3 gamma	*Eif2s3*	0.004	0.668
	Density-regulated protein	*Denr*	0.007	0.651
	Ribosomal protein L18A	***Rpl18a***	0.002	0.607
LIVER				
Up-regulated				
Fatty acid beta-oxidation (GO:0006635)	Acyl-Coenzyme A dehydrogenase, short/branched chain	*Acadsb*	0.010	0.922
	Acyl-Coenzyme A dehydrogenase, very long chain	*Acadvl*	0.001	0.842
	Hydroxyacyl-Coenzyme A dehydrogenase, beta subunit	*Hadhb*	0.008	0.711
	Hydroxyacyl-Coenzyme A dehydrogenase, alpha subunit	*Hadha*	0.007	0.586
	Acyl-Coenzyme A dehydrogenase, C-4 to C-12 straight chain	*Acadm*	0.001	0.510
Translation (GO:0006412)	Ribosomal protein L36	*Rpl36*	0.004	1.223
	Ribosomal protein L23	*Rpl23*	0.003	1.002
	Nascent polypeptide-associated complex alpha subunit	*Naca*	0.001	1.182
	Eukaryotic translation elongation factor 1 delta	*Eef1d*	0.001	1.170
	Translocated promoter region (to activated MET oncogene)	*Tpr*	<0.001	1.116
	Ribosomal protein S6	*Rps6*	0.010	1.079
	Ribosomal protein S20	*Rps20*	0.002	0.990
	Ribosomal protein L34	*Rpl34*	<0.001	0.929
	Ribosomal protein L9	*Rpl9*	0.005	0.829
	Ribosomal protein S16	*Rps16*	0.001	0.808
	Mediator complex subunit 8	*Med8*	0.004	0.683
	Ribosomal protein L28	*Rpl28*	0.001	0.617
	Protein kinase C, alpha	*Prkca*	0.004	0.572
	Isoleucyl-trna synthetase	*Iars*	0.007	0.565
Down-regulated				
Amino acid catabolic process (GO:0009063)	Arginase, liver	*Arg1*	0.009	-2.050
	Glutamic-oxaloacetic transaminase 1, soluble	*Got1*	0.003	-1.287
	Coenzyme A carboxylase, beta polypeptide	*Pccb*	<0.001	-1.176
	Aldehyde dehydrogenase 6 family, member A1	*Aldh6a1*	0.006	-1.086
	Methylcrotonoyl-Coenzyme A carboxylase 2 (beta)	*Mccc2*	0.001	-1.052
	Leucine aminopeptidase 3	*Lap3*	0.008	-0.943
	Branched chain keto acid dehydrogenase E1, beta	*Bckdhb*	<0.001	-0.937
	Glutamic-oxaloacetic transaminase 2, mitochondrial	*Got2*	0.001	-0.819
	Aminoadipate aminotransferase	*Aadat*	0.008	-0.812
	Indoleamine 2,3-dioxygenase 2	*Indol1*	0.003	-0.810
	Yippee-like 5	*Ypel5*	<0.001	-0.670
	3-hydroxyisobutyryl-Coenzyme A hydrolase	*Hibch*	0.004	-0.657
Cholesterol metabolic process (GO:0008203)	Cytochrome P450, family 7, subfamily a, polypeptide 1	*Cyp7a1*	0.001	-2.838
	Farnesyl diphosphate synthase)	*Fdps*	<0.001	-1.204
	Acetyl-Coenzyme A acetyltransferase 2	*Acat2*	0.001	-1.166
	Cytochrome P450, family 27, subfamily A, polypeptide 1	*Cyp27a1*	0.001	-1.082
	Paraoxonase 1	*Pon1*	0.005	-0.708
	7-dehydrocholesterol reductase	*Dhcr7*	0.001	-0.658
	Emopamil binding protein (	*Ebp*	0.020	-0.463
Cellular respiration (GO:0045333)	Cytochrome c, somatic	*Cycs*	0.002	-1.139
	Mitochondrial ribosomal protein S35	*Mrps35*	0.004	-0.859
	Coenzyme Q5 homolog, methyltransferase	*Coq5*	0.001	-0.783
	NADH dehydrogenase (ubiquinone) Fe-S protein 1	*Ndufs1*	0.003	-0.640
	Aconitase 1	*Aco1*	0.001	-0.627
	Succinate dehydrogenase complex, subunit C	*Sdhc*	0.004	-0.617
	Pyruvate dehydrogenase (lipoamide) beta	*Pdhb*	0.008	-0.558

Symbols of genes changed in both liver and muscle are in bold.

**Figure 2:**
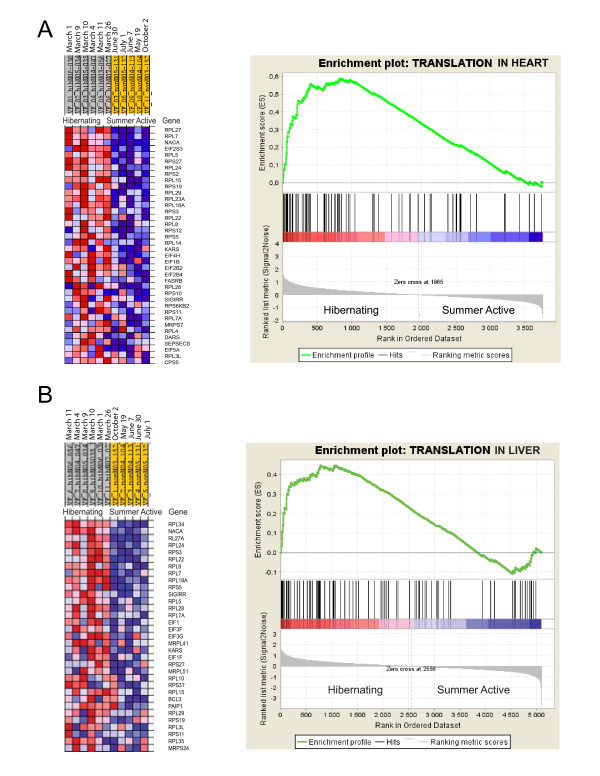
**Gene set enrichment analysis results for the translation category**. The translation category is enriched by up regulated genes in heart (A, false discovery rate (FDR) of <0.001) and liver (B, FDR = 0.046) of hibernating black bears. An expression data set sorted by correlation with hibernating phenotype and the corresponding heat map with red for up regulated and blue for down regulated genes during hibernation are shown on the left. Dates on the top indicate time of tissue sampling from each bear. Plot of the running sum for enrichment score (ES) in the data set (top) and location of genes (hits) from each GO category in the list ranked according to expression differences (middle) and the ranked list metric (bottom) are shown on the right.

**Figure 3:**
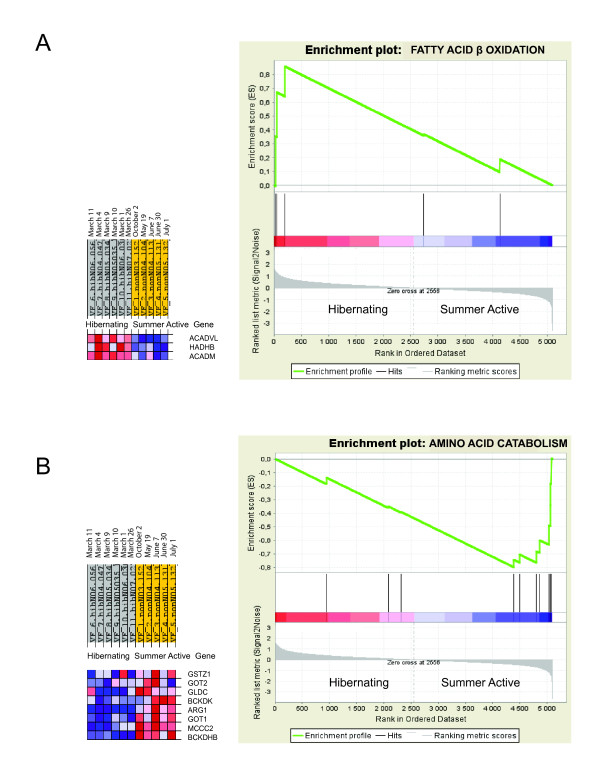
**Gene set enrichment analysis results for the fatty acid β oxidation and amino acid catabolism categories**. The fatty acid β oxidation category (A) is enriched by up regulated genes (FDR = 0.043) and amino acid catabolism (B) is enriched by down regulated genes (FDR = 0.015) in liver of hibernating black bears. The figure legend is explained in the text of Figure 2.

Significant enrichment of the biological processes categories by differentially expressed genes was validated and supported by the results of gene set enrichment analysis (GSEA; Figures 2, 3). GSEA ranks all genes with significant signals on the array therefore its results are not affected by the selection of genes above cutoffs for significance of expression differences and false discovery rate [13].

### Differential expression of selected genes in liver

Some important genes involved in metabolic pathways demonstrated expression differences between hibernating and summer active bears. Among 116 genes involved in lipid biosynthesis, expression of diacylglycerol O-acyltransferase (*Dgat2*), 7-dehydrocholesterol reductase (*Dhcr7*), emopamil binding protein (*Ebp*), farnesyl diphosphate synthase (*Fdps *), propionyl Coenzyme A carboxylase (*Pccb*) all decreased during winter hibernation. GSEA revealed significant enrichment (FDR = 0.006) of the lipid biosynthesis category by genes with lower expression levels in hibernating bears as compared to summer active animals. Expression of long-chain fatty acyl elongase (*Elovl2*) involved in biosynthesis of long chain fatty acids for catabolism through β oxidation increased during winter hibernation. Expressions of phosphoenolpyruvate carboxykinase (*Pck1*) and fructose-1,6-bisphosphatase 1 (*Fbp1 *), main control enzymes in the regulation of gluconeogenesis, were elevated during hibernation. In contrast, an important glycolytic gene, pyruvate dehydrogenase beta (*Pdhb*) and transketolase (*Tkt*) involved in the pentose phosphate pathway were down regulated in liver during hibernation.

Genes involved in protein catabolism did not show coordinated transcriptional change during hibernation. Among 140 genes involved in protein catabolism, leucine aminopeptidase 3 (*Lap*), calpastatin (*Cast*), proteasome 26S subunit (*Psmc2*) and proteasome subunit, beta type (*Psmb1*) were down regulated while YME1L1 gene (*Yme1l1*), ubiquitin protein ligase E3A (*Ube3a*), ubiquitin specific peptidase 7 (*Usp7*) and F-box and leucine-rich repeat protein 4 (*Fbxl4*) were over expressed in liver during hibernation.

### Differential expression of selected genes in heart

In common with liver (Table 2), there were three over expressed genes (*Acadvl, Acadm, Hadha*) that are involved in fatty acid beta oxidation in the heart of hibernating bears. Among 44 genes involved in amino acid catabolism, three genes shared with liver (*Aldh6a1*, *Got1*, *Lap3*) and glutathione transferase zeta 1 (*Gstz1*) were under expressed in heart during hibernation.

Although five out of 190 genes involved in protein catabolism were up regulated in heart during hibernation, similar to liver, this category did not demonstrate significant enrichment (FDR = 1.00). Over expressed protein catabolism genes included selenoprotein S (*Sels*), ornithine decarboxylase antizyme 1 (*Oaz1*), proteasome (prosome, macropain) 26S subunit (*Psmc3*), proteasome (prosome, macropain) subunit alpha type 3 (*Psma3*) ubiquitin specific peptidase 11 (*Usp11*).

## Discussion

Hibernating bears were sampled in March after at least 4 months of continuous hibernation and while in the absence of food and water. In Alaska black bears begin to emerge from hibernation in late April [14], thus, hibernating bears in this study were 3-6 weeks from emergence and the beginning of their return to summer levels of metabolism. All bears included as summer active or hibernating animals showed physiology and behavior that was expected for bear during summer and winter seasons, respectively.

Anesthesia was necessary for transport of the bears from their den and it was associated with a slight increase in metabolic rate compared to that measured in their dens. However, anesthesia used for immobilization of hibernating bears increased heart rate to the level observed in summer bears.

Black bears demonstrate a balanced proportion of up (52%) and down (48%) regulated genes that are differentially expressed in liver during hibernation. This contrasts to hibernating ground squirrels where 90% of differentially expressed genes are down-regulated in liver during hibernation [4]. This finding may reflect a greater level of homeostatic activity that is required by livers of hibernating bears compared to ground squirrels, since bears overwinter while maintaining relatively higher body temperatures and rates of metabolism than do small mammal hibernators [15-17]. The proportion of differentially expressed genes up-regulated during hibernation was even higher (75%) in bear heart and this is consistent with increased proportion (48%) of over expressed genes in heart of ground squirrels during torpor [18]. Increase in the transcription level for a number of genes in heart comparing to liver seems to be the general trend in the transcriptional changes for hibernating mammals.

### Protein biosynthesis and catabolism of nitrogen compounds

It has been recently shown that the coordinated induction in transcriptional level of protein biosynthesis genes in liver and skeleton muscle is a distinctive feature of the transcriptome in hibernating black bears comparing to small mammalian hibernators [11]. This conclusion is supported by the present study that identifies elevated expression of 19 protein biosynthesis genes in addition to 28 translation genes over expressed in liver during hibernation [11]. Similar to liver, we found elevated expression of 32 protein biosynthesis genes that generate significant enrichment of the translation category in heart during hibernation. Molecular function category of RNA binding is significantly enriched by over expressed genes in both liver and heart as RNA-binding proteins positively regulate the translation of RNA. Elevated expression of protein biosynthesis genes implies induction of translation in liver and heart during hibernation. A net increase in plasma protein concentration was found in hibernating brown and black bears [19,20], and this supports elevated protein synthesis in liver during hibernation. Bears have a unique ability to preserve muscle mass [8,21] and retain strength [22] through prolonged periods of inactivity and starvation during hibernation. The induction of protein biosynthesis in liver was suggested as molecular adaptation that contributes to ability to reduce muscle atrophy over prolonged periods of immobility during hibernation [11]. Decrease in protein synthesis was shown to be the main factor responsible for starvation-induced cardiac atrophy in non-hibernating mammals [23]. Transitional changes detected in our study imply the induction of protein biosynthesis in the heart of hibernating black bears and suggest an adaptive mechanism that reduces cardiac atrophy during prolonged fasting.

Comparing to other genes in the heart of hibernating bears, RNA binding motif protein 3 (*Rbm3*) demonstrated the maximum induction in expression. This cold induced RNA binding protein is the only gene consistently over expressed across different tissues in hibernating ground squirrels [4-6] and black bears [11]. It was suggested that RNA binding protein 3 protects mRNA transcripts in hibernating ground squirrels [4,6]. Taking into account coordinated induction of protein synthesis genes found in our study, over-expression of this protein biosynthesis gene may promote translation in heart of hibernating black bears. There is evidence showing that *Rbm3 *facilitates global protein synthesis under mild hypothermia at 32°C by binding to 60S ribosomal subunits and lowering abundance of microRNAs that dampen translation in human cell lines [24]. Over-expression of *Rbm3 *was suggested as part of compensatory mechanism to preserve mass of muscle undergoing disuse atrophy [25].

Under the condition of prolonged fasting, protein anabolism is directly related to metabolism of amino acids. Our results revealed coordinated down regulation of genes involved in amino acid catabolism and transaminase activity during hibernation. This finding implies a reduction in amino acid catabolism in hibernating black bears. Reduction in catabolism of amino acids is consistent with coordinated under expression of genes involved in amino group utilization through the urea cycle previously reported in hibernating bears [11] and significant decrease in the urea concentration in blood that was repeatedly observed during hibernation [21,26,27]. Under no dietary intake of amino acids, reduced amino acid catabolism and urea production suggest redirection of amino acids from catabolic pathways to enhanced protein biosynthesis.

### Fuel shift, cellular respiration and detoxification in liver

We found a coordinated induction of genes involved in fatty acid β oxidation in liver during hibernation. These transcriptional changes are consistent with physiological data showing that hibernating bears primarily use energy stored in fat [20], and this is further supported by respiratory quotient values near 0.7 that we observed in our hibernating bears [11]. In contrast, genes involved in lipid biosynthesis were down regulated in liver, as bears fasted throughout winter. Similar to black bears, evidence for induction of lipid catabolism were reported at transcriptional [4,6] and proteomic levels [28] in hibernating ground squirrels. In relation with lipid metabolism, our study also revealed transcriptional suppression of genes involved in cholesterol metabolism in liver during hibernation. The two cytochrome P450 genes (*Cyp7a1 *and *Cyp27a1*) catalyzing the first reaction in the cholesterol catabolic pathway in the liver, which converts cholesterol to bile acids, and oxidizing cholesterol intermediates were both down regulated during hibernation. Reduction in the cholesterol catabolism is supported by the elevation in cholesterol serum level that has been consistently observed in hibernating black bears [9,29].

A shift from glucose catabolism to glucose synthesis that provides an energy source for brain and other tissues in fasting conditions was observed at the mRNA and protein levels in liver of hibernating ground squirrels [6,28]. Similar to small mammalian hibernators, we detected over expression of the two key glucogenic enzymes (*Pck1*, *Fbp1*) as well as down regulation of important glycolytic (*Pdhb*) and the pentose phosphate shunt (*Tkt*) enzymes in liver during hibernation. These results taken together with under expression of glucokinase (*Gck*) catalyzing the irreversible step in glycolysis [11] suggest induction of glucose synthesis and reduction of glucose catabolism in liver of hibernating bears. Glycolysis is the first step of cellular respiration. Genes involved in cellular respiration demonstrated coordinated under expression in liver during hibernation. Apart from glycolytic enzymes, down regulated cellular respiration genes include key enzymes (*Sdhc*, *Aco1*) of the tricarboxylic acid cycle and genes involved in electron transport (*Cycs*, *Coqs*, *Ndufs1*). Molecular function category of electron carrier activity is significantly enriched by under expressed genes in liver as part of cellular respiration and oxidoreductation. Coordinated down regulation of cellular respiration genes in the liver, which plays the central role in metabolic homeostasis is consistent with the reduction of metabolic rate (20-50%) in hibernating bears.

Detoxification is an important function of the liver. We detected coordinated repression of genes involved in oxidoreductase activity, acting on the aldehyde or oxo group of donors and monooxygenase activity categories of molecular function. In these categories down regulated genes include a number of cytochrome P450 genes, flavin containing monooxygenases, aldehyde dehydrogenases that catalyze the oxidation of potentially toxic xenobiotics and metabolites with electron-deficient carbon centers (electrophilic compounds). These transcriptional changes are similar to expression differences detected in small hibernators [4,6], and they imply that detoxification function of the liver is depressed during hibernation, probably as a result of prolonged food deprivation.

## Conclusions

Elevated expression of multiple protein biosynthesis genes is a prominent feature of the transcriptome of hibernating black bears in all organs studied to date in black bears. Induction of protein synthesis may be related to adaptive mechanisms reducing cardiac and muscle atrophies over extended periods of low metabolism and immobility during hibernation in bears. Transcriptional reduction of genes involved in amino acid catabolism suggests redirection of amino acids from catabolic pathways to elevated protein biosynthesis.

We inferred functional changes by comparing transcripts abundance between hibernating and summer active bears. Due to post transcriptional regulatory mechanisms, protein abundance can be different from corresponding gene expression on the mRNA level [6,28]. However, functional significance of transcriptional changes detected in our study for the most important groups of co-regulated genes such as protein biosynthesis, catabolism of nitrogenous compounds and lipid metabolism is supported by independent lines of evidence coming from physiology and biochemistry of hibernating bears. Another point of support is that mammals generally demonstrate surprisingly high correlation between gene expression at mRNA and protein levels. The most comprehensive survey to date in model species revealed significant positive correlation between transcript and corresponding protein quantities for 71.4% of genes [30]. It is also notable that for another mammalian hibernator, significant correlation (Pearson's r = 0.62; P < 0.001) was found between expression on the mRNA and protein levels when comparing summer active and hibernating arctic ground squirrels [28]. Ongoing shotgun proteomic analysis will further validate functional significance of transcriptional changes reported here, identify regulatory changes undetectable on transcript level and, thus, provide more understanding of the molecular basis of hibernation in bears.

## Methods

### Animals

We analyzed heart and liver tissue from black bears that were reported on in a previous study [11]. Bears (31-143 kg) were captured May-July from the field in Alaska. Bears were held individually in a shaded outdoor holding facility in Fairbanks. In order to diminish effects of gender and age on intra-group variation in gene expression, only males > 2 years old were used in these experiments. Non-hibernating bears (n = 5) were feeding and active when they were euthanized and sampled for tissues between late May and early October. We stopped feeding bears 24 hours before these animals were euthanized. Hibernating bears were sacrificed for tissue sampling between 1-27 March (n = 6), about one month before their expected emergence from hibernation. These animals were without food since October 27. Animal protocols were approved by the University of Alaska Fairbanks Institutional Animal Care and Use Committee (protocols no. 02-39, 02-44, 05-55, 05-57) and USAMRMC Animal Care and Use Review Office (proposal Number 05178001).

### Physiological monitoring and tissue harvesting

For monitoring of physiological conditions, bears were instrumented as previously described [11]. Briefly, core body temperature, ECG and EMG was monitored with radio telemetry. Beginning in late November, bears were housed in individual outdoor enclosures on the University of Alaska Fairbanks campus that had dens and straw material for nests. Dens were closed with a break-away door, air was drawn though the dens, and oxygen consumption and RQ monitored with an open flow respirometry system. The procedure for tissue harvesting was a previously described [11]. On the day of tissue harvesting, bears were immobilized between 9:30-15:00 using Telazol (8-10 mg/kg) and transported to a necropsy suite in a nearby building. Oxygen consumption in immobilized bears was checked on a subsample of animals with an open flow respirometry system during blood sampling just prior to euthanasia via a tracheal tube, and ECG was recorded for at least 40s on a chart recorder or recorded at 400 Hz sampling frequency with data acquisition system (LabGraph with Scientific Solutions LAbmaster TM40-PGL board). Between the first disturbance of bears and the beginning of tissue sampling 40-65 minutes elapsed. Bears were euthanized by an intravenous injection of pentobarbital with death assessed by termination of heart beats as assessed with a stethoscope. Tissues were sampled immediately and frozen in liquid nitrogen within 12 min. Heart tissue was sampled from the apex.

### RNA preparation

RNA was extracted from frozen tissues stored at -80°C by using RNeasy Kit (Qiagen). All RNA samples were treated with DNase I (Qiagen). RNA quality was assessed with an Agilent 2100 Bioanalyzer and concentration was measured by using Nanodrop ND-1000.

### Developing genomic resources

Normalized, subtracted cDNA libraries were constructed from brain, liver, testis, heart and skeletal muscle. We used SMART template-switching protocol and primer extension PCR [31], normalization and subtraction [32] as described [11]. Details of the cDNA libraries constructed for this study are available at the Black Bear Gene Index Project [33]. Expressed sequence tags (EST) were sequenced from the 5'-end with the universal M13 forward primer. After filtering off low quality reads, vector contamination and mtDNA inserts, 38,328 high-quality ESTs were clustered and then assembled into 4,925 unique Tentative Consensus (TC) sequences and 12,719 singleton ESTs using the TCICL software package [34] and annotated by searching against a non-redundant protein database. The ESTs and resulting TC sequences used in this study are available from the Black Bear Gene Index, release 4.0 [32]. Genomic analysis of the bear EST collection was reported elsewhere [12]. In addition to the first bear array (BA01) containing 3,200 probes [11], 9,600 unique annotated cDNA inserts were PCR amplified and printed on the second bear array (BA02) with a Biorobotics arrayer in the Microarray core facility, the Wistar Institute [35].

### Hybridization

Total of 11 heart RNA samples from six hibernating and five summer active bears were hybridized in the same experiment with the two bear arrays (BA01; BA02 corresponding to GPL8249 and GPL13263 platforms in Gene Expression Omnibus database) that contain total of 12,800 cDNA probes. Six hibernating and five summer active bears RNA liver samples were hybridized with the 9,600 probe BA02 only as results for liver from the 3,200 BA01 had been reported elsewhere [11]. Samples of total RNA were linearly amplified with Illumina TotalPrep RNA Amplification Kit (Ambion), labeled with [^33^P]dCTP and hybridized with array filters as previously described [11,36]. All RNA samples from one tissue were amplified, labeled and hybridized in the same batch. After hybridization and washing filters were exposed to phosphorimager screens for four days and scanned at 50-μm resolution in a Storm Phosphorimager. Images were analyzed by using the ImaGene program (Biodiscovery).

### Microarray data analysis

Only spots with no flags were included in the analysis. Background correction was done by subtracting local background median density from signal median density. Background corrected signals were divided by their median on the array to obtain the normalized median densities representing the normalized expression values. One-way ANVOA test was used to select genes that exhibited significant differences between hibernating and summer active bears. A p-value < 0.01 and |log_2 _fold change| > 0.5 were set as cutoffs for significant different expressed genes, corresponding to the mean false discovery rate (FDR) around 10 - 13%. The FDR was calculated using random permutation as described by Storey and Tibshirani [37]. The FDR was defined as the number of significant selected genes divided by the average of the number of significant genes under permutations. The genes demonstrating significant hybridization signal on the arrays were classified according to their Gene Ontology (GO) categories of the biological processes and molecular functions. For each probe with a significant expression value, a gene identifier was obtained by blast search of the probe EST against the NCBI human RefSeq Data Base [38]. When a blast score was ≥100 the probe was assigned to that corresponding human gene symbol. For ESTs that mapped to more than one human reference sequence, we selected the hit with the highest blast score. If one gene had multiple probes on the array, we selected the probe with the smallest ANOVA p-value to represent the gene. Lists of all significant genes on the array and differentially expressed genes were loaded to GO miner [39]. The enrichment in each GO category was estimated as the proportion of differentially expressed genes relative to the expected proportion on the array. The GO gene sets with fewer than five differentially expressed genes detected were omitted from the analysis [40]. Significant GO categories with the same members have been selected on the basis of the GO hierarchical tree structure: if a parent term and its child term were significantly enriched by the same set of differentially expressed genes, narrow and more specific child category rather than broad parent term was considered. The significance of enrichment for each GO category was estimated by one-sided Fisher exact test. The false discovery rate was assessed by resampling the total significant genes on the array [18,40]. In addition to GO miner analysis, we verified enrichment in significant GO categories of the biological processes by using Gene Set Enrichment Analysis [41]. Unlike GO miner, GSEA estimates enrichment by taking into account all of the genes with significant signals in an experiment without constrain of arbitrary cutoffs for significance of expression differences, false discovery rate and fold-change [13]. Genes were ranked according to the extent of the correlation between their expression values and the phenotype class (hibernating or summer active) by using the signal to noise ratio. We calculated an enrichment score (ES) that reflects the degree to which genes involved in category are overrepresented at the extremes (top or bottom) of the entire ranked list of genes. The false discovery rate was estimated by using phenotype-based permutation test [13]. All microarray data were submitted to NCBI Gene Expression Omnibus (GEO) with accession number GSE27875.

### Quantitative real-time PCR

We validated the microarray experiments by 352 quantitative real-time PCR tests using the same total RNA samples. Nineteen genes were validated in liver and 13 genes in heart tissue. *Prmt1 *was selected as a reference gene for heart based on the stability of expression values across all samples obtained from the microarray experiments and then tested by RT PCR. All bear samples showed similar expression values with low standard deviation in multiple RT-PCR tests. *Hnrpf *was used as a reference gene for liver [11]. Conditions for cDNA synthesis, primer design (Additional file 1, Table S2) and real time PCR amplifications were described elsewhere [11]. Negative control RT PCRs with no template were taken to exclude contamination, and controls with no reverse transcriptase but all other components were taken to exclude false amplification from genomic DNA. Specificity of amplification was checked with the melting curve analysis and agarose gel electrophoresis. Four 10-fold dilutions of a sample with mixed cDNA were used for a standard curve for each primer set for calculating RT PCR efficiency. We calculated the fold-change in level of expression of a target gene relative to a reference gene for each sample and then compared the values for hibernating and summer active bears using Student's *t*-test [42,43].

## Authors' contributions

BMB and VBF conceived the study. ØT collected physiological data and tissue samples. AVG, NCS, CC, LCS and MKS designed and conducted expression experiments. AVG, HW, VBF and JY analyzed data. VBF, BMB, ØT, and LCS wrote the paper. All authors read and approved the final manuscript.

## Supplementary Material

Additional file 1 **Supplementary tables**. **Table S1 **List of differentially expressed genes identified in this study. Listed genes demonstrate significant expression differences between hibernating and summer active black bears in heart and liver tissues. Genes are ranked by log_2_FC (Fold Change), P is a significance level. Positive significant genes are up-regulated (positive values of log_2_FC) and negative significant genes are down-regulated (negative values of log_2_FC) in hibernating animals. Complete description of the black bear EST collection can be found in The Black Bear Gene Index at http://compbio.dfci.harvard.edu/tgi/cgi-bin/tgi/T_release.pl?gudb=bear. **Table S2 **Primer sequences used for the real-time PCR tests in this study. Click here for file
