# Supplementary material for: Modulation of gene expression in heart and liver of hibernating black bears (Ursus americanus)
Source: BMC Genomics. 2011 Mar 31;12:171. doi: 10.1186/1471-2164-12-171 (PMC3078891; doi:10.1186/1471-2164-12-171)
Supplement: Additional file 1 — Supplementary tables. Table S1 List of differentially expressed genes identified in this study. Listed genes demonstrate significant expression differences between hibernating and summer active black bears in heart and liver tissues. Genes are ranked by log2FC (Fold Change), P is a significance level. Positive significant genes are up-regulated (positive values of log2FC) and negative significant genes are down-regulated (negative values of log2FC) in hibernating animals. Complete description of the black bear EST collection can be found in The Black Bear Gene Index at http://compbio.dfci.harvard.edu/tgi/cgi-bin/tgi/T_release.pl?gudb=bear. Table S2 Primer sequences used for the real-time PCR tests in this study. [file 1471-2164-12-171-S1.DOC]

**Additional file 1, Table S1**

| Gene Symbol | Clone ID | | P value | Log_2_FC |
| --- | --- | --- | --- | --- |
| HEART |  | |  |  |
| *Rbm3* | 008_031_J09 | | <0.001 | 2.863 |
| *Kiaa0528* | 05202004A_000_011B_C10_06 | | 0.004 | 2.725 |
| *Otud1* | 007_009_B15 | | 0.004 | 2.487 |
| *Slc38a2* | 007_003_A18 | | 0.001 | 2.078 |
| *Cyr61* | 010-001A_C12_2007-03-20 | | <0.001 | 2.003 |
| *Ahnak* | 007_024_O07 | | 0.002 | 1.987 |
| *Rpl35a* | 005-020C_E07_2006-11-11 | | <0.001 | 1.857 |
| *Rps23* | 007_020_H08 | | 0.001 | 1.793 |
| *Btg2* | 008_028_N09 | | 0.001 | 1.778 |
| *Prtfdc1* | 008_007_G05 | | 0.009 | 1.442 |
| *Pcbd1* | 008_027_K08 | | 0.006 | 1.409 |
| *Sh3rf2* | 005_005_I14 | | 0.003 | 1.358 |
| *Ywhaz* | 013-002_H16 | | 0.002 | 1.347 |
| *Rps12* | 003_002A_F05 | | 0.004 | 1.307 |
| *Tead1* | 007_031_B15 | | 0.004 | 1.287 |
| *Rpl7a* | 004_003C_C12 | | 0.002 | 1.287 |
| *Aasdhppt* | 005_006_A08 | | 0.004 | 1.255 |
| *Mansc1* | 005-020C_H02_2006-11-10 | | 0.005 | 1.251 |
| *Rpl7* | 005-014C_B05_2006-10-05 | | <0.001 | 1.243 |
| *Setd3* | 005-015C_A06_2006-10-20 | | 0.001 | 1.196 |
| *Commd3* | 005-022D_D10_2006-11-23 | | 0.003 | 1.190 |
| *Eef1b2* | UAhib_1708.y1_001 | | 0.001 | 1.187 |
| *Parl* | 008_012_D12 | | <0.001 | 1.180 |
| *Rpl14* | 001_002A_H08 | | 0.004 | 1.175 |
| *Rps3* | 004_002A_E05 | | 0.005 | 1.166 |
| *Rpl10a* | 004_001A_B03 | | 0.003 | 1.156 |
| *Eif5a* | 007_005_C22 | | <0.001 | 1.147 |
| *Rps18* | 008B-001C_E08_2006-11-09 | | 0.002 | 1.144 |
| *Acadm* | 005_006_O23 | | <0.001 | 1.138 |
| *Sap30* | 005-019C_F04_2006-12-01 | | 0.001 | 1.126 |
| *Rps2* | 004_001D_E10 | | 0.002 | 1.125 |
| *Rps15a* | 008-001C_G01_2006-11-07 | | 0.008 | 1.116 |
| *Cox17* | 001_002B_D08 | | 0.001 | 1.114 |
| *Gnb2l1* | 004_006B_F09 | | 0.002 | 1.112 |
| *Ehbp1* | 008_011_B17 | | 0.003 | 1.107 |
| *Rpl27* | 004_003D_B08 | | 0.003 | 1.099 |
| *Cited2* | 007_022_I20 | | 0.002 | 1.085 |
| *Sels* | 007_014_C18 | | 0.005 | 1.080 |
| *Prg4* | UAhib_0838.y1_037 | | 0.001 | 1.077 |
| *C10orf83* | 005_006_H09 | | 0.008 | 1.077 |
| *Kars* | 004_001C_D02 | | <0.001 | 1.075 |
| *Mnat1* | 005-017B_G11_2006-11-07 | | 0.006 | 1.067 |
| *B3galnt1* | 008_026_E05 | | <0.001 | 1.062 |
| *Ifrd1* | 007_001_B16 | | 0.003 | 1.059 |
| *Blcap* | 003_001C_G07 | | 0.001 | 1.054 |
| *Hnrnpa1* | 008B-001A_E12_2006-10-31 | | 0.006 | 1.048 |
| *Hnrpd* | 008_014_I15 | | <0.001 | 1.028 |
| *Gpi* | UAhib_0468.y1_073 | | 0.002 | 1.024 |
| *Rps4x* | 011-015_I11 | | 0.001 | 1.018 |
| *Rps13* | 005-014B_F11_2006-11-03 | | 0.003 | 1.012 |
| *Glrx3* | 005_007_M03 | | 0.001 | 1.011 |
| *Ndufa11* | 004_002D_G08 | | 0.008 | 1.011 |
| *Rpl30* | 007_024_C23 | | 0.002 | 1.010 |
| *Rps24* | 007_015_L22 | | 0.002 | 1.000 |
| *Fxr1* | 008_009_G04 | | <0.001 | 0.996 |
| *Hint1* | 004_003D_F03 | | 0.006 | 0.994 |
| *Atp5l* | 001_002B_D07 | | 0.001 | 0.983 |
| *Sync1* | 005_001A_B12_B12_2006-05-12 | | 0.009 | 0.980 |
| *Cct7* | 007_031_F02 | | <0.001 | 0.976 |
| *St7* | 005_010_G16 | | 0.003 | 0.965 |
| *Gapdh* | 005-016C_F06_2006-11-03 | | 0.008 | 0.963 |
| *Suclg2* | 008B-001A_C08_2006-10-30 | | 0.002 | 0.950 |
| *Atp6v0d1* | 05152004A_000_010D_C07_05 | | 0.004 | 0.940 |
| *Rplp0* | 008_003_F03 | | 0.001 | 0.936 |
| *Txnip* | 011-015_N19 | | 0.006 | 0.930 |
| *Rpl27a* | 014-004_F02 | | 0.008 | 0.927 |
| *Rpl22* | 005-022D_H12_2006-11-23 | | 0.004 | 0.923 |
| *Dnajb2* | 003_003B_G02 | | 0.006 | 0.921 |
| *Rpl36al* | 011-010_C01 | | 0.005 | 0.917 |
| *Hif1an* | 005-021C_D02_2006-12-05 | | 0.003 | 0.915 |
| *Uba52* | 008_029_H18 | | 0.009 | 0.915 |
| *Loc100133169* | 005-023A_B08_2006-11-09 | | 0.004 | 0.913 |
| *Myeov2* | 008_030_J02 | | 0.006 | 0.911 |
| *Eif3g* | 007_008_G16 | | 0.001 | 0.910 |
| *Golga7* | 008_004_E06 | | 0.007 | 0.908 |
| *Ccdc98* | 005-019C_C02_2006-12-01 | | 0.008 | 0.902 |
| *C11orf58* | 005_003_F16 | | 0.001 | 0.901 |
| *Rpl5* | 005-018A_F11_2006-10-13 | | 0.002 | 0.900 |
| *Rbbp4* | 005-014A_F09_2006-11-02 | | <0.001 | 0.893 |
| *Slc5a6* | 007_003_M18 | | 0.006 | 0.891 |
| *Tnpo1* | 007_030_N14 | | 0.001 | 0.885 |
| *Mbip* | 008_003_C21 | | 0.001 | 0.884 |
| *Eif3f* | 013-006_I04 | | 0.003 | 0.884 |
| *Smarcc2* | 007_029_A21 | | 0.003 | 0.881 |
| *C1orf181* | 007-019D_A09_2006-11-01 | | 0.001 | 0.879 |
| *Mrlc2* | 007_010_J09 | | 0.001 | 0.879 |
| *Med13l* | 03312004A_000_007B_A02_02 | | 0.003 | 0.870 |
| *Serpine2* | 007_020_E10 | | 0.004 | 0.868 |
| *Myh2* | 011-009_L11 | | 0.003 | 0.864 |
| *Rpl10* | 011-013_H23 | | 0.008 | 0.863 |
| *Arl1* | 007_011_N08 | | 0.004 | 0.851 |
| *Ubxn1* | 008_010_M06 | | 0.005 | 0.846 |
| *Rps29* | 011-016_J20 | | 0.001 | 0.843 |
| *Msrb3* | 005_011_N19 | | 0.005 | 0.842 |
| *Fdft1* | 004_001C_F11 | | 0.005 | 0.841 |
| *Gbe1* | 013-007_K20 | | 0.002 | 0.838 |
| *Aarsd1* | 008_004_D04 | | 0.006 | 0.838 |
| *Akap11* | 007_001_O15 | | 0.007 | 0.831 |
| *Cdk2* | 008_014_C09 | | 0.005 | 0.827 |
| *Prg-3* | 014-004_G07 | | <0.001 | 0.827 |
| *Sfrs10* | 01082004_000_001_B11_B11_03 | | 0.008 | 0.821 |
| *Stk19* | 007_031_E12 | | 0.004 | 0.818 |
| *Tacc2* | 004_002B_F09 | | 0.004 | 0.817 |
| *Tinp1* | 005-017C_G03_2006-10-19 | | <0.001 | 0.813 |
| *Churc1* | 008_014_L20 | | 0.008 | 0.812 |
| *Ormdl3* | 007-019C_D04_2006-10-17 | | <0.001 | 0.802 |
| *Psmc3* | 007_013_J23 | | <0.001 | 0.778 |
| *Mn1* | 007_030_N17 | | 0.001 | 0.776 |
| *Hmgn2* | 007_008_I10 | | 0.004 | 0.775 |
| *Pkig* | 005-012B_H06_2006-11-17 | | 0.005 | 0.768 |
| *Oaz1* | 008B-001C_E02_2006-11-08 | | 0.002 | 0.764 |
| *Lmcd1* | 011-015_F23 | | 0.004 | 0.761 |
| *Rnh1* | 008_003_C18 | | 0.005 | 0.757 |
| *Setd5* | 007_002_J21 | | 0.004 | 0.756 |
| *Spag7* | 005_003_G07 | | 0.003 | 0.756 |
| *Snurf* | 03312004A_000_007B_B06_04 | | 0.005 | 0.748 |
| *Acadvl* | 008_021_H08 | | <0.001 | 0.747 |
| *Psip1* | 008_029_I08 | | 0.003 | 0.743 |
| *Tcf4* | 005-019D_C01_2006-11-14 | | 0.008 | 0.740 |
| *Rplp2* | 005_007_A08 | | 0.001 | 0.732 |
| *Dis3l* | 008_009_A21 | | 0.003 | 0.728 |
| *Rpl24* | 008B-001B_G01_2006-10-28 | | 0.003 | 0.725 |
| *Acaa2* | UAhib_1530.y1_022 | | 0.006 | 0.724 |
| *Rps28* | 011-005_M08 | | 0.009 | 0.724 |
| *Rps27* | 005-012D_G04_2006-12-12 | | 0.001 | 0.720 |
| *Gpd1* | 008-001C_F04_2006-11-07 | | <0.001 | 0.712 |
| *Vps28* | 007_004_D08 | | 0.007 | 0.703 |
| *Dnajc8* | 008_004_O05 | | <0.001 | 0.698 |
| *Psma3* | 010-001B_E09 | | 0.006 | 0.698 |
| *Loc202051* | 005-025C_G05_2006-12-08 | | 0.002 | 0.696 |
| *Rnf113a* | 007_004_E19 | | <0.001 | 0.690 |
| *Gabarapl2* | 007_008_K16 | | <0.001 | 0.688 |
| *Cops6* | 005-013C_D09_2006-12-09 | | <0.001 | 0.687 |
| *Tcea3* | 011-015_D09 | | 0.005 | 0.682 |
| *Smarca1* | 007_031_E24 | | 0.005 | 0.681 |
| *Wdr23* | 008_012_G19 | | <0.001 | 0.679 |
| *Zcchc17* | 008_024_K12 | | 0.008 | 0.677 |
| *Eif2s3* | 007_004_H15 | | 0.004 | 0.668 |
| *Rac1* | 005-002A_D01_2006-08-18 | | 0.009 | 0.656 |
| *Brap* | 008_004_L09 | | 0.007 | 0.653 |
| *Denr* | 007_008_B07 | | 0.007 | 0.651 |
| *Hnrph2* | 007_010_P14 | | 0.001 | 0.649 |
| *Klhdc3* | 005-012A_C03_2006-11-15 | | 0.005 | 0.649 |
| *Usp11* | 007_012_P19 | | 0.003 | 0.649 |
| *Fus* | 007_011_A04 | | <0.001 | 0.644 |
| *Rragb* | 007_008_J16 | | 0.005 | 0.644 |
| *Loc645899* | 008B-001A_H06_2006-10-30 | | 0.007 | 0.639 |
| *Tnnc2* | 011-002C_F06 | | 0.006 | 0.635 |
| *Nola1* | 007_031_C12 | | 0.006 | 0.632 |
| *Gapdh* | 007_006_J23 | | 0.008 | 0.620 |
| *Bmp1* | 008_006_D19 | | 0.001 | 0.616 |
| *Cd2bp2* | 007_007_A10 | | 0.008 | 0.614 |
| *Znf19* | 005-013D_A08_2006-11-18 | | 0.003 | 0.608 |
| *Rpl18a* | 005-016D_A01_2006-11-10 | | 0.002 | 0.607 |
| *Terf2ip* | 007_023_P03 | | 0.005 | 0.604 |
| *Rnf10* | 007_023_J17 | | 0.005 | 0.599 |
| *Zeb2* | 007_007_E11 | | 0.004 | 0.593 |
| *C5orf33* | 008-001D_D05_2006-11-07 | | 0.007 | 0.590 |
| *Add3* | 008_019_D10 | | 0.003 | 0.587 |
| *Phldb3* | 010-003A_E10 | | 0.002 | 0.583 |
| *Atp5g2* | 005-019D_G02_2006-11-14 | | <0.001 | 0.579 |
| *Bhmt2* | 008_014_L22 | | 0.001 | 0.578 |
| *Klf10* | 008_023_G04 | | 0.009 | 0.578 |
| *Rbms1* | 011-015_B13 | | 0.001 | 0.576 |
| *Acly* | 05012004_000_009C_A03_01 | | 0.004 | 0.567 |
| *Gmps* | 011-008_P15 | | 0.003 | 0.566 |
| *Gnai3* | 005_010_A02 | | 0.007 | 0.558 |
| *Hadha* | 005-013C_E04_2006-12-09 | | 0.008 | 0.556 |
| *Dhrs7* | 005-022A_E10_2006-11-29 | | <0.001 | 0.556 |
| *Bbs2* | 007_020_L07 | | 0.002 | 0.555 |
| *Atp6v1c1* | 007_033_K08 | | 0.005 | 0.555 |
| *Fgg* | UAhib_1869.y1_071 | | 0.006 | 0.554 |
| *Ppp1r7* | 005-022C_H04_2006-11-22 | | 0.003 | 0.554 |
| *Ewsr1* | 008_008_K13 | | 0.001 | 0.544 |
| *Thra* | 007_015_C21 | | 0.005 | 0.541 |
| *Tarp* | 008_028_F14 | | 0.006 | 0.534 |
| *Clk1* | 008_029_A15 | | 0.009 | 0.534 |
| *Hnrpk* | 007_031_I08 | | 0.009 | 0.531 |
| *Hnrnpa2b1* | 007-019B_G08_2006-10-19 | | 0.005 | 0.531 |
| *Fbxl5* | 010-003B_G08 | | 0.006 | 0.526 |
| *Sap18* | 005-012C_D07_2006-11-28 | | 0.008 | 0.508 |
| *C6orf151* | 005-002D-Anya_E12_2006-08-25 | | 0.008 | 0.505 |
| *Hebp1* | 007_013_A10 | | 0.007 | 0.500 |
| *Kif21a* | 007_024_J07 | | 0.009 | -0.504 |
| *Serf2* | 005-017A_B06_2006-11-07 | | 0.008 | -0.507 |
| *Mat1a* | 008_008_C06 | | 0.005 | -0.507 |
| *Tmem126a* | 007_013_M02 | | 0.004 | -0.518 |
| *Nxph1* | 007_015_N06 | | 0.003 | -0.523 |
| *Mrvi1* | 013-002_N11 | | 0.001 | -0.527 |
| *Tcf25* | 008B-001D_B10_2006-11-08 | | 0.003 | -0.528 |
| *Adk* | 008_003_F13 | | 0.006 | -0.534 |
| *Fam65a* | 005-022A_A04_2006-11-29 | | 0.006 | -0.537 |
| *Rnf7* | 011-011_B07 | | 0.009 | -0.538 |
| *Dync1h1* | 007_006_C08 | | 0.005 | -0.541 |
| *Ccdc132* | 007_006_P21 | | 0.008 | -0.545 |
| *Agl* | 011-004_G09 | | <0.001 | -0.557 |
| *Ergic1* | 008_032_L04 | | 0.003 | -0.558 |
| *Uchl5ip* | 007_011_I12 | | 0.003 | -0.562 |
| *Calm3* | 008_006_F22 | | 0.001 | -0.564 |
| *Cenpj* | 007_021_P04 | | 0.007 | -0.568 |
| *Cyp1a2* | 008_030_C16 | | 0.005 | -0.594 |
| *Ckmt2* | 005-016A_B10_2006-11-03 | | 0.009 | -0.598 |
| *Ube2v1* | 007_014_L18 | | 0.004 | -0.600 |
| *Gstz1* | 008_006_O06 | | 0.001 | -0.605 |
| *Ift140* | 007_033_L07 | | 0.007 | -0.611 |
| *Iqgap1* | 008_031_M20 | | 0.007 | -0.612 |
| *Ppm1k* | 005-015B_E01_2006-10-20 | | 0.007 | -0.615 |
| *Pou6f2* | 007_006_K05 | | 0.004 | -0.624 |
| *Tubgcp5* | 007_022_O03 | | 0.009 | -0.624 |
| *Ivd* | 005-023D_E05_2006-11-16 | | 0.004 | -0.630 |
| *Pnpla8* | 007_026_K15 | | 0.007 | -0.634 |
| *Med30* | 007_021_A05 | | 0.002 | -0.638 |
| *Got1* | 007_003_A04 | | 0.002 | -0.644 |
| *Ltbr* | 005-012B_H09_2006-11-17 | | 0.008 | -0.658 |
| *Optn* | 007_008_M05 | | 0.006 | -0.669 |
| *Mus81* | 007B-001A_B11_2006-10-19 | | 0.003 | -0.682 |
| *Opa1* | 007_022_F24 | | 0.001 | -0.687 |
| *Gpr172b* | 007_004_G12 | | 0.003 | -0.692 |
| *Tmem33* | 007_004_P15 | | 0.002 | -0.693 |
| *Atp5j2* | 006-001A_G10_2006-10-10 | | 0.003 | -0.694 |
| *Mafg* | 007_028_L12 | | 0.003 | -0.694 |
| *Adssl1* | 005_009_P02 | | 0.006 | -0.724 |
| *Tgfbi* | 008_015_I08 | | 0.010 | -0.726 |
| *Apba2* | 007_007_J19 | | 0.003 | -0.745 |
| *Hsd17b10* | 005-012B_C10_2006-11-17 | | 0.008 | -0.755 |
| *Znf292* | 007_010_M07 | | 0.003 | -0.761 |
| *Ces3* | 006-001A_A10_2006-10-10 | | 0.002 | -0.767 |
| *Lap3* | 007_022_F01 | | 0.001 | -0.771 |
| *Loc100130890* | 007_007_L11 | | 0.003 | -0.819 |
| *Rab10* | 007_004_O06 | | 0.010 | -0.821 |
| *B4galnt1* | 007_006_C06 | | 0.005 | -0.845 |
| *Katnal1* | 007_006_D15 | | 0.002 | -0.846 |
| *Zfp106* | 008_007_N03 | | <0.001 | -0.850 |
| *Phyh* | 008_010_D04 | | 0.008 | -0.865 |
| *Aldh6a1* | 008_016_G18 | | 0.001 | -0.893 |
| *Fuca1* | 008_031_N14 | | <0.001 | -0.910 |
| *Xirp2* | 005-024B_E09_2006-11-15 | | <0.001 | -0.980 |
| *Rgs5* | 008_028_I02 | | 0.002 | -1.018 |
| *Hagh* | 008_025_C21 | | <0.001 | -1.061 |
| *Sobp* | 007_014_G15 | | 0.001 | -1.150 |
| *Gucy1a2* | 007_009_C13 | | 0.001 | -1.371 |
| *Ube2q2* | 005-021C_F08_2006-12-05 | | 0.005 | -1.384 |
| *Ank1* | 005-016A_B12_2006-11-03 | | <0.001 | -1.440 |
| *Acot8* | 007_004_D23 | | 0.003 | -1.593 |
| *Smyd2* | 005-018B_E09_2006-10-13 | | 0.003 | -1.841 |
| LIVER |  | |  |  |
| *Pck1* | | 007_020_P23 | <0.001 | 3.426 |
| *Rbm3* | | 008_031_J09 | <0.001 | 2.681 |
| *Loc728003* | | 008_012_E18 | 0.002 | 2.598 |
| *Lepr* | | 008-001A_E12_2006-11-02 | <0.001 | 2.449 |
| *Gpc3* | | 008_032_N02 | <0.001 | 1.777 |
| *Rplp2* | | 005_007_A08 | 0.003 | 1.627 |
| *Bhlhb2* | | 008B-001D_G10_2006-11-08 | 0.002 | 1.593 |
| *Cmtm8* | | 011-014_L14 | <0.001 | 1.525 |
| *Hsd17b13* | | 008_029_M22 | 0.001 | 1.459 |
| *Hnrnpa1* | | 005_006_M16 | 0.004 | 1.418 |
| *Rpl22* | | 005-022D_H12_2006-11-23 | 0.006 | 1.398 |
| *Pc* | | 007_022_J22 | 0.002 | 1.390 |
| *Loc730004* | | 011-008_O06 | 0.003 | 1.368 |
| *Loc100129857* | | 008_027_D04 | 0.001 | 1.353 |
| *Rpl27a* | | 014-004_F02 | 0.004 | 1.349 |
| *Elovl2* | | 008_015_L18 | 0.001 | 1.299 |
| *Crip2* | | 007_010_M15 | 0.001 | 1.285 |
| *A2m* | | 008_005_K17 | 0.004 | 1.249 |
| *Rpl36* | | 006-001B_E08_2006-10-11 | 0.004 | 1.223 |
| *Rps23* | | 007_020_H08 | <0.001 | 1.213 |
| *Brp44l* | | 007_007_A17 | 0.009 | 1.198 |
| *Naca* | | 005-015C_A04_2006-10-20 | 0.001 | 1.182 |
| *Eef1d* | | 011-005_D12 | 0.001 | 1.170 |
| *Tfb1m* | | 008_020_D15 | 0.009 | 1.165 |
| *Tmem17* | | 010-003D_A05 | <0.001 | 1.161 |
| *Fgfr2* | | 007_024_P18 | <0.001 | 1.130 |
| *Tbca* | | 005-014A_B05_2006-11-02 | 0.001 | 1.122 |
| *Tpr* | | 007_010_L14 | <0.001 | 1.116 |
| *Ppm1k* | | 005-015B_E01_2006-10-20 | 0.005 | 1.107 |
| *Brp44* | | 014-004_F24 | <0.001 | 1.083 |
| *Rps6* | | 007_029_K10 | 0.010 | 1.079 |
| *Narg1* | | 008_008_B08 | <0.001 | 1.070 |
| *Nsun2* | | 008_013_P13 | 0.001 | 1.067 |
| *Gnas* | | 005-016C_A11_2006-11-03 | 0.002 | 1.061 |
| *Hnrnpa0* | | 007-019D_H10_2006-11-01 | 0.002 | 1.060 |
| *Fgg* | | 008-001C_G03_2006-11-07 | 0.003 | 1.049 |
| *Trib1* | | 008_012_A05 | 0.001 | 1.038 |
| *Loc100130553* | | 008B-001C_E08_2006-11-09 | 0.007 | 1.023 |
| *Pros1* | | 008_029_P13 | 0.001 | 1.012 |
| *Rpl23* | | 011-006_E18 | 0.003 | 1.002 |
| *Kif21a* | | 007_002_D16 | 0.010 | 1.000 |
| *Ppap2b* | | 007_009_C02 | 0.001 | 0.995 |
| *Rps20* | | 007_009_C16 | 0.002 | 0.990 |
| *Slc17a4* | | 008_013_N05 | 0.006 | 0.989 |
| *Adh4* | | 008_010_M21 | 0.002 | 0.988 |
| *Maged1* | | 007_029_G15 | <0.001 | 0.986 |
| *Cd2bp2* | | 007_007_A10 | 0.005 | 0.981 |
| *Rps3* | | 011-011_A13 | 0.004 | 0.979 |
| *Cfb* | | 008_016_G02 | 0.003 | 0.973 |
| *Fndc3b* | | 008_005_J07 | 0.007 | 0.972 |
| *Loc100129608* | | 005-024A_F06_2006-11-14 | 0.007 | 0.954 |
| *Fbxl4* | | 008_016_N07 | <0.001 | 0.947 |
| *Rab1a* | | 008_015_O17 | 0.006 | 0.944 |
| *Rpl35a* | | 005-020C_E07_2006-11-11 | 0.001 | 0.937 |
| *Rpl24* | | 011-001A_G04 | 0.005 | 0.935 |
| *Cntnap2* | | 007_006_L22 | 0.005 | 0.933 |
| *Rarres2* | | 005-024C_C12_2006-12-01 | <0.001 | 0.932 |
| *Rpl34* | | 008_013_D04 | <0.001 | 0.929 |
| *Ptp4a1* | | 006-001A_H05_2006-10-10 | 0.006 | 0.928 |
| *Fgb* | | 008_015_B14 | <0.001 | 0.928 |
| *Acadsb* | | 008-001C_A10_2006-11-08 | 0.010 | 0.922 |
| *Trappc2l* | | 005_001A_B01_B01_2006-05-12 | 0.002 | 0.921 |
| *Klhl23* | | 005-021B_G08_2006-12-07 | 0.004 | 0.915 |
| *Loc647030* | | 007_024_P02 | 0.003 | 0.911 |
| *St7* | | 005_010_G16 | 0.001 | 0.909 |
| *Lgr4* | | 007_011_P21 | 0.002 | 0.897 |
| *Cyp20a1* | | 008_016_N08 | 0.006 | 0.868 |
| *Btbd10* | | 007_010_N19 | <0.001 | 0.864 |
| *Rpl30* | | 007_024_C23 | 0.004 | 0.862 |
| *Tspan12* | | 005-023D_F05_2006-11-16 | 0.002 | 0.860 |
| *Mat2a* | | 005-022A_D07_2006-11-29 | <0.001 | 0.860 |
| *H3f3b* | | 005-022C_A07_2006-11-22 | 0.001 | 0.860 |
| *Rab13* | | 008_028_K13 | 0.002 | 0.850 |
| *Mtmr10* | | 008_005_D15 | <0.001 | 0.845 |
| *Acadvl* | | 008_021_H08 | 0.001 | 0.842 |
| *Krtcap2* | | 011-014_N06 | 0.002 | 0.842 |
| *Ttc31* | | 008-001B_B10_2006-10-27 | 0.002 | 0.840 |
| *Clk3* | | 008_011_N23 | 0.003 | 0.840 |
| *Loc100130862* | | 008_006_A03 | 0.004 | 0.839 |
| *Art3* | | 005_010_L23 | 0.001 | 0.837 |
| *Agxt2l1* | | 006-001A_D01_2006-10-10 | 0.004 | 0.836 |
| *Vapa* | | 007_010_H01 | 0.004 | 0.832 |
| *Rpl9* | | 008_003_H16 | 0.005 | 0.829 |
| *Mapk1ip1l* | | 008_009_D04 | 0.007 | 0.820 |
| *Ddx19a* | | 007_024_K20 | 0.004 | 0.811 |
| *Rps16* | | 011-006_H22 | 0.001 | 0.808 |
| *Uxt* | | 008_015_D19 | 0.003 | 0.807 |
| *Prg4* | | 008_007_F07 | 0.009 | 0.803 |
| *Rps24* | | 007_015_L22 | 0.008 | 0.802 |
| *Fbp1* | | 005_009_I11 | 0.007 | 0.802 |
| *Loc642892* | | 011-016_J20 | 0.006 | 0.802 |
| *Dnai1* | | 008_008_K22 | 0.006 | 0.796 |
| *Pdgfc* | | 007_013_E23 | 0.008 | 0.794 |
| *Narg1l* | | 008_019_O09 | <0.001 | 0.792 |
| *Rxra* | | 008_023_N15 | 0.001 | 0.789 |
| *Serpinf2* | | 008_032_A10 | 0.009 | 0.789 |
| *Polr3gl* | | 007_021_D15 | <0.001 | 0.786 |
| *C17orf61* | | 005-021B_F03_2006-12-06 | 0.006 | 0.784 |
| *Tuba1a* | | 007_021_P07 | 0.002 | 0.784 |
| *Pkp2* | | 008_007_E15 | 0.007 | 0.776 |
| *Arid5b* | | 008-001A_F06_2006-11-02 | 0.003 | 0.776 |
| *Rpl7* | | 007_023_H24 | 0.008 | 0.767 |
| *Tmem19* | | 008_016_C06 | 0.005 | 0.767 |
| *Asph* | | 008_008_F21 | 0.004 | 0.767 |
| *Arpc3* | | 007_033_J02 | 0.010 | 0.757 |
| *Cdk2* | | 008_014_C09 | 0.001 | 0.755 |
| *Hadhb* | | 005-013C_B12_2006-12-09 | 0.003 | 0.749 |
| *Wdr26* | | 008B-001D_C07_2006-11-08 | 0.007 | 0.748 |
| *Usp7* | | 008_012_D18 | <0.001 | 0.733 |
| *Ehbp1* | | 008_011_B17 | 0.003 | 0.733 |
| *Prpf40a* | | 007_014_B14 | 0.005 | 0.730 |
| *Hspc152* | | 005-016D_C07_2006-11-10 | <0.001 | 0.727 |
| *Pglyrp2* | | 008-001C_C02_2006-11-07 | 0.006 | 0.725 |
| *C11orf10* | | 005-013D_C02_2006-11-18 | 0.005 | 0.724 |
| *Dnajc8* | | 008_004_O05 | <0.001 | 0.720 |
| *C14orf166* | | 007_011_B03 | 0.007 | 0.720 |
| *Fpgs* | | 007_011_C07 | 0.004 | 0.710 |
| *Hspe1* | | 007_030_J12 | 0.007 | 0.693 |
| *C19orf53* | | 008_009_H02 | 0.001 | 0.693 |
| *Mettl7b* | | 008_022_N10 | 0.001 | 0.683 |
| *Med8* | | 011-013_N20 | 0.004 | 0.683 |
| *Usf2* | | 005-018D_H05_2006-10-25 | 0.002 | 0.683 |
| *Mag* | | 007_005_L22 | <0.001 | 0.678 |
| *Tinp1* | | 005-017C_G03_2006-10-19 | 0.010 | 0.678 |
| *Rnf128* | | 007-019C_F02_2006-10-17 | 0.001 | 0.671 |
| *Rcor3* | | 008_015_N23 | 0.009 | 0.665 |
| *Ppp2r1b* | | 007_027_I05 | 0.001 | 0.645 |
| *Atp5a1* | | 005_009_C11 | 0.002 | 0.642 |
| *Aarsd1* | | 005_011_O04 | 0.006 | 0.638 |
| *Vamp8* | | 005_011_J08 | 0.007 | 0.635 |
| *Ccdc76* | | 008_023_K08 | 0.006 | 0.635 |
| *Myom2* | | 005_006_M10 | 0.002 | 0.631 |
| *Ptpn11* | | 007_013_K14 | 0.002 | 0.624 |
| *Yme1l1* | | 005_007_A07 | 0.005 | 0.623 |
| *Rpl28* | | 007_008_F08 | 0.001 | 0.617 |
| *Pdhx* | | 005-016D_E08_2006-11-10 | 0.005 | 0.610 |
| *Rcn1* | | 008_022_E07 | <0.001 | 0.606 |
| *Rpl18a* | | 005-016D_A01_2006-11-10 | <0.001 | 0.606 |
| *Idh2* | | 005-020d_H03_2006-12-02 | 0.002 | 0.598 |
| *Gmnn* | | 007_021_C22 | 0.010 | 0.595 |
| *Pnpla4* | | 008_029_G11 | 0.006 | 0.593 |
| *Myo1b* | | 008_014_E02 | 0.001 | 0.587 |
| *Sept10* | | 005-023C_A11_2006-11-16 | 0.006 | 0.587 |
| *Hadha* | | 005-013C_E04_2006-12-09 | 0.007 | 0.586 |
| *Tm9sf2* | | 008_006_N10 | 0.001 | 0.578 |
| *Ube3a* | | 007_023_F03 | 0.002 | 0.577 |
| *Lmo4* | | 011-013_I10 | 0.004 | 0.573 |
| *Prkca* | | 007_007_J08 | 0.004 | 0.572 |
| *Pigw* | | 007_031_J03 | 0.005 | 0.568 |
| *Iars* | | 008_020_H10 | 0.007 | 0.565 |
| *C6orf70* | | 007_008_J08 | <0.001 | 0.560 |
| *Gnpnat1* | | 008_005_O10 | 0.007 | 0.558 |
| *Cpne1* | | 007_002_D03 | 0.009 | 0.556 |
| *Rpl27* | | 005-012A_F11_2006-11-15 | 0.007 | 0.546 |
| *Siva1* | | 007_003_H13 | 0.004 | 0.538 |
| *Kif1b* | | 007-019B_B01_2006-10-19 | <0.001 | 0.531 |
| *Fgf11* | | 007_011_K17 | 0.007 | 0.520 |
| *Nop5/Nop58* | | 011-013_K22 | 0.004 | 0.518 |
| *Sfrs11* | | 007_012_B07 | <0.001 | 0.517 |
| *Lrrc2* | | 005_010_I02 | 0.010 | 0.516 |
| *Acadm* | | 005_006_O23 | 0.001 | 0.510 |
| *Pfn2* | | 005_010_D03 | 0.009 | 0.506 |
| *Slu7* | | 007_001_N04 | 0.005 | 0.505 |
| *Slc37a3* | | 008_005_A18 | 0.009 | 0.505 |
| *Arhgap5* | | 007_008_C03 | 0.008 | 0.504 |
| *Pla1a* | | 008_013_A03 | 0.002 | -0.502 |
| *Pus7l* | | 008_011_D24 | 0.006 | -0.505 |
| *Gapvd1* | | 008_012_C11 | 0.006 | -0.505 |
| *Pabpc4* | | 008_003_O17 | 0.007 | -0.507 |
| *Mterfd1* | | 008_006_F10 | 0.002 | -0.507 |
| *Cap2* | | 007_006_K22 | 0.009 | -0.508 |
| *Rnf14* | | 007_001_F10 | 0.004 | -0.511 |
| *Tmem165* | | 007_003_K23 | 0.003 | -0.514 |
| *Pqbp1* | | 005_010_K04 | 0.001 | -0.516 |
| *C21orf63* | | 007_027_L24 | 0.004 | -0.529 |
| *Thap6* | | 008_031_I17 | 0.008 | -0.533 |
| *C9orf86* | | 008_019_F18 | 0.006 | -0.535 |
| *Itga1* | | 008_025_J09 | 0.005 | -0.535 |
| *Loc728599* | | 007_007_O20 | <0.001 | -0.542 |
| *Gpi* | | 005-019B_E03_2006-10-12 | 0.003 | -0.542 |
| *Rnft1* | | 008_007_O21 | 0.009 | -0.544 |
| *Cd81* | | 007_024_B10 | 0.001 | -0.546 |
| *Ube2v2* | | 008_027_C10 | 0.002 | -0.550 |
| *Ppp1ca* | | 005-012A_D01_2006-11-15 | 0.005 | -0.550 |
| *Heatr2* | | 008_014_D20 | 0.004 | -0.551 |
| *Pdhb* | | 007_014_E03 | 0.008 | -0.558 |
| *Fgf1* | | 007_005_P06 | 0.009 | -0.559 |
| *Aldh1a1* | | 008_008_I20 | 0.001 | -0.569 |
| *Mrps5* | | 008_028_D04 | <0.001 | -0.573 |
| *Tsfm* | | 005-015C_F10_2006-10-21 | <0.001 | -0.576 |
| *M6pr* | | 011-010_B03 | 0.002 | -0.586 |
| *U1snrnpbp* | | 005-012C_B02_2006-11-28 | 0.001 | -0.587 |
| *Glrx3* | | 005_007_M03 | 0.004 | -0.595 |
| *Plekhb2* | | 005-020A_C11_2006-11-11 | 0.007 | -0.601 |
| *Aldh2* | | 008_005_L07 | 0.010 | -0.606 |
| *Atp6v1a* | | 007_004_K07 | 0.002 | -0.609 |
| *Amz2* | | 008_012_N16 | 0.003 | -0.610 |
| *Rcl1* | | 008_005_N15 | 0.004 | -0.611 |
| *Fos* | | 005-021A_B08_2006-11-01 | 0.006 | -0.615 |
| *Gdpd2* | | 008_026_I24 | 0.008 | -0.616 |
| *Sdhc* | | 007_012_J03 | 0.004 | -0.617 |
| *Ranbp3l* | | 007_002_C02 | 0.009 | -0.620 |
| *Dimt1l* | | 005-019D_B03_2006-11-14 | 0.002 | -0.620 |
| *Znf77* | | 005-015A_C05_2006-10-25 | 0.002 | -0.622 |
| *Vhl* | | 005-018D_A09_2006-10-25 | 0.006 | -0.626 |
| *Aco1* | | 008_027_D21 | 0.001 | -0.627 |
| *F7* | | 008_006_P04 | 0.004 | -0.637 |
| *Ndufs1* | | 005-025B_H03_2006-10-14 | 0.003 | -0.640 |
| *Gbe1* | | 013-007_K20 | 0.007 | -0.641 |
| *Znrf1* | | 007_003_K02 | 0.002 | -0.648 |
| *Hibch* | | 007_009_J22 | 0.004 | -0.657 |
| *Arl8b* | | 005_004_F22 | 0.003 | -0.657 |
| *Tmem9b* | | 007_010_E02 | 0.001 | -0.658 |
| *Dhcr7* | | 008-001D_H03_2006-11-07 | 0.001 | -0.658 |
| *Gckr* | | 008_015_I18 | 0.001 | -0.664 |
| *Txndc1* | | 008_009_M20 | 0.001 | -0.666 |
| *Ypel5* | | 005_007_C15 | <0.001 | -0.670 |
| *Sepx1* | | 005_003_D22 | 0.001 | -0.674 |
| *Mnat1* | | 005-015D_F04_2006-10-26 | 0.007 | -0.675 |
| *Ces7* | | 008_008_H08 | 0.001 | -0.676 |
| *Elof1* | | 007_007_G03 | 0.002 | -0.696 |
| *Ifitm1* | | 008_029_F13 | 0.002 | -0.697 |
| *Fuca2* | | 007_030_C15 | 0.004 | -0.704 |
| *Pon1* | | 006-001A_B04_2006-10-10 | 0.005 | -0.708 |
| *Prdx6* | | 007_005_M10 | 0.007 | -0.709 |
| *C11orf31* | | 005-014D_H07_2006-11-21 | 0.006 | -0.714 |
| *Map7d3* | | 005_008_K21 | 0.001 | -0.719 |
| *Sult1a4* | | 008_018_L13 | 0.004 | -0.721 |
| *Pten* | | 005_010_O19 | 0.003 | -0.724 |
| *Rps6ka1* | | 007_027_P12 | 0.003 | -0.726 |
| *Znf706* | | 008_004_J14 | 0.001 | -0.727 |
| *Tm6sf2* | | 008_022_J24 | 0.009 | -0.739 |
| *Csda* | | 005_011_E02 | 0.002 | -0.750 |
| *Prdm2* | | 007_008_H07 | 0.003 | -0.751 |
| *B2m* | | 005-016D_G02_2006-11-10 | 0.006 | -0.754 |
| *Cast* | | 005-023D_D01_2006-11-16 | 0.004 | -0.758 |
| *Scarb2* | | 007_025_A14 | 0.006 | -0.775 |
| *Cyp2d6* | | 008_003_H23 | 0.001 | -0.775 |
| *Mtch2* | | 007_014_C13 | <0.001 | -0.780 |
| *Coq5* | | 005-020d_B08_2006-12-02 | 0.001 | -0.783 |
| *Map2k3* | | 007_003_D21 | 0.001 | -0.784 |
| *Loc642384* | | 005_007_F08 | 0.005 | -0.801 |
| *Capza1* | | 008_006_M10 | 0.005 | -0.808 |
| *Foxo3* | | 007-019B_E10_2006-10-19 | 0.008 | -0.808 |
| *Indol1* | | 008_004_D14 | 0.003 | -0.810 |
| *Snx3* | | 007_001_B02 | <0.001 | -0.812 |
| *Aadat* | | 008_029_P05 | 0.008 | -0.812 |
| *Sft2d2* | | 008_028_M22 | 0.008 | -0.817 |
| *Got2* | | 013-006_F09 | 0.001 | -0.819 |
| *Mgc39900* | | 007_008_M24 | 0.010 | -0.823 |
| *Slc22a1* | | 008_013_O23 | 0.006 | -0.824 |
| *Phc1* | | 007_009_I23 | <0.001 | -0.829 |
| *Gpsn2* | | 008-001C_H05_2006-11-07 | <0.001 | -0.830 |
| *G3bp1* | | 005-022B_G02_2006-11-30 | <0.001 | -0.836 |
| *Sord* | | 007_023_I16 | 0.001 | -0.838 |
| *Tkt* | | 008_023_B09 | 0.007 | -0.840 |
| *Psmb1* | | 005_010_I23 | 0.001 | -0.842 |
| *Mrps35* | | 005-020C_H06_2006-11-10 | 0.004 | -0.859 |
| *Mphosph6* | | 008_024_E08 | 0.004 | -0.871 |
| *Wac* | | 008_010_C13 | 0.008 | -0.888 |
| *Papss2* | | 008_019_H09 | 0.007 | -0.907 |
| *Sept7* | | 005_007_H14 | 0.002 | -0.907 |
| *Uox* | | 008_013_P22 | 0.003 | -0.909 |
| *C8g* | | 008_006_M17 | 0.008 | -0.925 |
| *Gabrb3* | | 007_007_E22 | 0.002 | -0.926 |
| *Agxt* | | 008B-001D_C05_2006-11-08 | 0.002 | -0.929 |
| *Bckdhb* | | 008_010_O12 | <0.001 | -0.937 |
| *Lap3* | | 007_008_H15 | 0.008 | -0.943 |
| *Acadl* | | 008_007_C13 | 0.006 | -0.946 |
| *Sult1a1* | | 007_013_F15 | 0.006 | -0.947 |
| *Slc25a1* | | 007_030_P15 | <0.001 | -0.948 |
| *C9orf103* | | 008_011_E08 | 0.001 | -0.958 |
| *Arl6ip1* | | 007_030_O13 | 0.001 | -0.980 |
| *Glyat* | | 008_009_D16 | 0.010 | -0.995 |
| *Mgst1* | | 008_013_I09 | 0.001 | -1.004 |
| *Fam14a* | | 007_002_K12 | 0.005 | -1.013 |
| *Got1* | | 007_025_D05 | <0.001 | -1.014 |
| *Cnot10* | | 008_013_G23 | <0.001 | -1.029 |
| *Dgat2* | | 007_014_K18 | 0.005 | -1.036 |
| *Mccc2* | | 005-022B_D06_2006-11-30 | 0.001 | -1.052 |
| *Sv2a* | | 007_002_I02 | 0.010 | -1.085 |
| *Aldh6a1* | | 008_016_G18 | 0.006 | -1.086 |
| *Lamp2* | | 005-023C_F06_2006-11-15 | 0.003 | -1.088 |
| *Ebp* | | 005-019D_D07_2006-11-14 | <0.001 | -1.090 |
| *Slc25a11* | | 005-015C_G09_2006-10-21 | 0.010 | -1.117 |
| *Slc35d1* | | 008_006_N24 | 0.001 | -1.118 |
| *Cyp3a4* | | 006-001A_H06_2006-10-10 | 0.004 | -1.124 |
| *Ugp2* | | 008_024_M02 | 0.001 | -1.129 |
| *Cycs* | | 007_004_C01 | 0.002 | -1.139 |
| *Alas1* | | 007_005_M14 | 0.004 | -1.152 |
| *Cbr1* | | 008B-001A_G12_2006-10-31 | 0.001 | -1.154 |
| *Acat2* | | 007_029_E06 | 0.001 | -1.166 |
| *Erp29* | | 005_008_H24 | 0.003 | -1.170 |
| *Pccb* | | 007_010_P19 | <0.001 | -1.176 |
| *Sar1a* | | 008_018_F20 | 0.005 | -1.188 |
| *Nap1l1* | | 007_008_O14 | <0.001 | -1.201 |
| *Dhrs1* | | 011-008_E06 | <0.001 | -1.203 |
| *Fdps* | | 007_010_C20 | <0.001 | -1.204 |
| *Nav2* | | 007_012_E14 | 0.005 | -1.216 |
| *Fkbp5* | | 005-024A_F03_2006-11-14 | <0.001 | -1.254 |
| *Loc642103* | | 008_016_I03 | <0.001 | -1.334 |
| *Acot12* | | 008_005_C15 | 0.006 | -1.406 |
| *Dhdh* | | 008_016_H10 | <0.001 | -1.456 |
| *Cps1* | | 008_025_A05 | <0.001 | -1.457 |
| *Cyp27a1* | | 008_031_P08 | <0.001 | -1.699 |
| *Psmc2* | | 005-021C_B09_2006-12-05 | 0.001 | -1.707 |
| *Fmo1* | | 008_015_G14 | 0.003 | -1.800 |
| *Comt* | | 007_027_L06 | 0.001 | -1.817 |
| *Slc1a2* | | 007_013_F01 | 0.003 | -1.843 |
| *Igsf6* | | 005-021C_C03_2006-12-05 | <0.001 | -1.851 |
| *Arg1* | | 008_020_A09 | 0.009 | -2.050 |
| *Phyh* | | 008_010_D04 | 0.004 | -2.105 |
| *Tinag* | | 008_003_L22 | <0.001 | -2.141 |
| *Cyp1a2* | | 008_030_C16 | 0.007 | -2.290 |
| *Cyp7a1* | | 008_013_K07 | 0.001 | -2.838 |
| *Ugt3a2* | | 008_005_A02 | <0.001 | -2.949 |
| *Fmo5* | | 008_018_K17 | 0.002 | -3.002 |
| *Pitrm1* | | 007_013_A01 | 0.001 | -3.360 |
| *Aldh1l1* | | 008_003_G18 | <0.001 | -4.168 |

**Additional file 1, Table S2**

| GeneSymbol | Forward | Reverse |
| --- | --- | --- |
| *Acadvl* | ATGAGGGGCATCATTGCTAA | TCACGTACTGCAGCATAGCC |
| *Dnajc8* | CGGAACTTCCAAGCAAATAC | CACGCTGCTCCATTTTTAC |
| *Gapdh* | GAGGACCAGGTTGTCTCCTG | TGCTGTAGCCAAATTCGTTG |
| *Mbip* | CTGCTGCCGTTGAGCTTAGT | AGTTGTCCCACCAGGCTATG |
| *Rbm3* | TCAGCAGCTTCGGTCCTATT | TGGTGAAGGTGATGAAACCA |
| *Rpl35a* | AGGCAAGAGATGTGCTTATGT | GTTTCCATGAGCACGAGTTAC |
| *Rpl7* | GAATTGCCCTGACAGATAACA | AAACGTTTTCCAACAGTGTAGA |
| *Rps27* | CCCCAACTCCTACTTCATGG | GCAGCCGACACACAAAACTA |
| *Adk* | GGAGCTGGAGATGCATTTGT | ATAGTGGCCAGCACGGATAC |
| *Aldh6a1* | CCAACCTCATTTGCCAACTT | CAGGGGCTAATGGTCTTGAG |
| *Ank1* | AAGTGGCACACCTGTTCTCTA | GTCAGTCTTTCATGCGGATAG |
| *Gstz1* | GGTGTCCATGGCTGATCTCT | GGCCATTAGGGTCTTGTTGA |
| *Ube2v1* | CCTCGCAATTTTCGACTGTT | TTGGAGGCCCAATTATCATC |
| *Gpc3* | GAATCTCGGAATCGAGGTCA | AGTCCCTGGCAGTAAGAGCA |
| *Rplp2* | TGCCAAGGACATCAAGAAGA | TTCCGTTCAGCTCACTGATG |
| *Cmtm8* | TCATCGTTCTTCGCCTTTTT | GGTCCTGGATCTCCAAGCTA |
| *Pc* | TTCATCGTTCTTCGCCTTTT | TCCAAGCTATAAAACTGAAATATGTG |
| *Crip2* | AAGTGCGACAAGACCGTGTA | GCGCTCACACTTGAGACAGA |
| *A2m* | ATGACGGTGACAGGAGAAGG | CTTTGGGTCCGTCACAAGTT |
| *Rpl36* | GGACATGATCCGAGAGGTGT | GCTTGTCCTTGGAGACCTTG |
| *Brp44l* | TGACATTTGCCCTGTGTTGT | GAATGAGCTGGGCTACTTCG |
| *Naca* | ATTCAACACAGGCAACCACA | TCTTTTCACTCCGGCTCTGT |
| *Fgfr2* | GACCCGCTGTGGGTACTG | TCCAATTACTACAAAATTCACAAGGA |
| *Tbca* | CAGGTTGGAAGCTGCACATA | TCCAATACTAAACGTGCTTCCTT |
| *Rps6* | ACGATGAACGCAACTTCGTA | CCTTCCATTCTTCACCCAGA |
| *Comt* | GCCTGGTCTACATCCTCTGG | GATACGCTGCTCCTTGGTGT |
| *Arg1* | TAAACTGGGAATTGGCAAGG | GGGTCCAATCCATCAACATC |
| *Phyh* | ATGTGGAGTGCTTCACTGGA | TAGTGCAGATCCTGGTGCAA |
| *Tinag* | AATGGCCTCCTCACACAAAG | GCATCTCCATTGCTGTCTGA |
| *Ugt3a2* | GTGACAGAAGCTTTGCATGG | GCAAATCACTGCATCGAATC |
| *Fmo5* | TGCACTCCAATCCAGTATCG | ATCATGGGCTTCCTGATACG |
| *Pitrm1* | GTCTTTCTCGAAGGCTGTGG | GTCCAGCCCTCTGTCTGAAG |
| *Prmt1* | CGGCACCTCTTCAAGGATAA | CTGGAACACTCGATCCCAAT |
